# Effects of Caffeine and *Rhodiola rosea*, Alone and Combined, on DanceSport-Specific Jive Performance: A Randomized, Blinded, Placebo-Controlled Trial

**DOI:** 10.3390/nu18172823

**Published:** 2026-08-28

**Authors:** Xincheng Dai, Meiling Liu, Shuning Liu, Jiakai Tang, Longqi Yu, Chang Liu, Jingyan Yu, Xian Li, Tao Chou, Chang Liu

**Affiliations:** 1 School of Art, Beijing Sport University, Beijing 100084, China; 2 School of International Education and Exchange, Beijing Sport University, Beijing 100084, China; 3 School of Sports Science, Beijing Sport University, Beijing 100084, China; 4 Division of Sports Science and Physical Education, Tsinghua University, Haidian, Beijing 100084, China; 5 Department of Biostatistics, Columbia University Mailman School of Public Health, New York, NY 10032, USA; 6 College of the Competitive Sports, Beijing Sport University, Beijing 100084, China

**Keywords:** DanceSport, Jive, caffeine, *Rhodiola rosea*, valid-kick success rate, ANCOVA, randomized trial, ClinicalTrials.gov

## Abstract

Background/Objectives: Evidence on caffeine (CAF), *Rhodiola rosea*, and their combined use in DanceSport remains limited. This randomized, blinded, placebo-controlled trial, retrospectively registered at ClinicalTrials.gov (NCT07690085), evaluated Jive-specific performance after a four-week standardized intervention. Methods: Forty-eight male collegiate DanceSport athletes were randomly allocated by drawing lots to placebo control (CTR), CAF, RHO (*Rhodiola rosea*), or RHO + CAF (n = 12/group). RHO was administered using Tongrentang Brand Rhodiola Capsule (0.4 g/capsule; three capsules twice daily; 2.4 g·day^−1^) for four weeks, and CAF was administered at 3 mg·kg^−1^ body mass 30 min before Post testing. The registry lists four primary outcomes: kick-extension angle, post-kick leg-retraction angle, trunk anteroposterior sway, and 30 s valid-kick success rate. Post values were analyzed by ANCOVA using the corresponding Pre value as covariate, with Holm correction across the primary-outcome family. Results: Training attendance was 100%. All four primary-outcome tests remained significant after Holm correction (adjusted *p* = 0.000768–0.006182; partial η^2^ = 0.248–0.386). Adjusted success rates were 84.66% (CTR), 90.69% (CAF), 84.43% (RHO), and 92.31% (RHO + CAF); CAF and RHO + CAF were higher than CTR and RHO. The CAF × RHO interaction was not significant. Conclusions: Group differences were outcome-specific. The combined condition showed the highest adjusted success rate, but the data did not establish a synergistic interaction. The findings apply to the *Rhodiola rosea* intervention used in this study.

## 1. Introduction

The Latin discipline of DanceSport comprises five dances: Samba, Cha-Cha-Cha, Rumba, Paso Doble, and Jive. Compared with the other dances, Jive is characterized by a fast tempo, frequent bouncing actions, repeated kick-and-retraction transitions, and high demands on continuous lower-limb output [1,2]. Physiological profiling of elite dancers also supports substantial whole-body demands during dance performance [3]. During competitive routines or multiple-round events, athletes usually enter the Jive section after having accumulated fatigue from previous dances, yet they must still maintain repeated bouncing, kicking, leg retraction, weight transfer, and trunk control under rapid musical rhythm. Biomechanical and injury-prevention perspectives support evaluating movement quality and control beyond a single isolated action [4,5]. Strength-and-conditioning evidence likewise supports assessment of the physical capacities underlying dance performance [6]. Cardiorespiratory evidence indicates substantial physiological demands during dance training and performance [7,8]. DanceSport-specific studies also report athlete-like physiological and anthropometric characteristics in competitive performers [9,10].

In this framework, the Jive kick requires rapid forward extension while bringing the maximum knee-extension angle toward 180°. The technical thresholds used in this study were >175° for a valid kick and ≥178° for an excellent kick; values closer to 180° indicated more complete knee extension and better technical execution. This angle characterizes movement completion rather than kick velocity or explosive power. After extension, the leg must be retracted rapidly; a retraction angle ≥ 50° was considered acceptable and ≥60° represented a higher technical level. Trunk anteroposterior sway was evaluated concurrently, with lower values indicating better midline control. Athletes also had to maintain these criteria and musical timing across a 30 s task comprising 32 kicks. Together, these variables represent distinct components of Jive-specific execution.

Jive-specific performance depends on rapid initiation at the front end of the movement sequence and stable repeated execution during the maintenance phase. Caffeine (CAF) acts primarily through adenosine-receptor antagonism and is broadly recognized as an ergogenic aid across exercise contexts [11,12]. Meta-analytic evidence supports effects on endurance performance [13] and muscular strength/endurance [14]. Effects on muscle strength and power have also been reported [15]. Low-dose and inter-individual response considerations may modify caffeine ergogenicity [16,17]. Intermittent-sprint studies report performance benefits under some conditions [18,19]. Team-sport meta-analyses likewise indicate context-dependent effects [20,21]. Acute *Rhodiola rosea* studies have reported effects on endurance-related outcomes [22,23]. Reviews suggest that responses across fatigue and exercise-performance outcomes remain heterogeneous [24,25]. A sport-specific systematic review reached a similarly cautious conclusion [26]. More recent evidence includes constituent-specific and football studies [27,28]. A recent meta-analysis also indicates heterogeneity across endurance outcomes and related biomarkers [29]. In this manuscript, RHO denotes *Rhodiola rosea*. These mechanisms were not measured in the present study and are presented only as a physiological rationale. We hypothesized that CAF-containing conditions would most clearly affect repeated technical execution and rhythm-dependent outcomes, whereas RHO-related effects would be task-specific; any combined effect was evaluated formally rather than assumed to be synergistic.

Evidence concerning CAF, *Rhodiola rosea*, and combined CAF + RHO supplementation has mainly been derived from endurance, strength, team-sport, and general fitness tasks. A recent football trial examined *Rhodiola rosea*-related performance outcomes in a sport-specific setting [28]. Combined CAF + *Rhodiola rosea* studies have examined strength, muscular endurance, and aerobic-performance outcomes [30,31]. Additional work has extended this combined-supplement approach to volleyball and soccer performance [32,33]. However, cross-study comparison is limited by differences in product composition, dose expression, and standardization. Jive requires musical-rhythm control, movement-path accuracy, trunk stability, post-kick retraction, and sustained technical quality. Repeated kick-and-retraction is a fundamental and indispensable technical element of Jive, and the 30 s repeated-kick task directly evaluates the sustained quality of this element under standardized conditions. The remaining field tests do not reproduce the complete judged choreography; instead, they assess supporting physical capacities—lower-limb force production, core and balance control, rhythm maintenance, stop–restart ability, and multidirectional coordination—that underpin the quality and repeatability of Jive technique.

This study evaluated CAF, *Rhodiola rosea* (RHO), and combined supplementation during a four-week Jive-specific training intervention in male collegiate DanceSport athletes aged 18–23 years. The trial was retrospectively registered at ClinicalTrials.gov (NCT07690085; Unique Protocol ID: 2026252H). The registry lists kick-extension angle, post-kick leg-retraction angle, trunk anteroposterior sway amplitude, and 30 s valid-kick success rate as primary outcomes. Registry-listed secondary outcomes comprised valid-kick count, YBT composite balance, bird-dog core stability, split-lunge squat, step-up/box step, 45 s high-knee rhythm compliance, 505 change-of-direction time, Illinois agility time, hexagon-jump time, and session-RPE. Success rate was calculated at the participant level as valid-kick count/32 × 100.

## 2. Materials and Methods

### 2.1. Participants

This study included 48 male collegiate DanceSport athletes aged 18–23 years. All participants had more than 3 years of systematic DanceSport training, maintained regular Jive-specific training exposure, and were proficient in competitive Jive combinations and repeated kicking actions. All participants completed PAR-Q screening before formal testing. Within sport-science participant-classification frameworks, this cohort represents trained collegiate athletes and should not be equated with professional or international-level elite competitors [34].

Inclusion criteria were age 18–23 years; male collegiate status; at least 3 years of systematic DanceSport training; at least three specific training sessions per week; and health status sufficient to complete the four-week intervention and Pre/Post testing. Exclusion criteria were neuromuscular disease, severe cardiovascular disease, severe musculoskeletal injury, or a lower-limb/lumbar injury affecting testing; use of medication with a potential interaction with RHO or CAF; contraindication to either supplement; regular use of coffee, strong tea, energy drinks, other ergogenic supplements, alcohol, or tobacco within 3 months before enrollment; or inability to maintain stable training, diet, and sleep habits. Eligibility was confirmed by questionnaire and interview.

Before enrollment, the research team explained the study purpose, procedures, study-related risks, and supplementation requirements and obtained written informed consent. During the intervention, participants were instructed to maintain their habitual diet and training habits, avoid additional sports-nutrition supplements, ergogenic substances, and caffeine-containing foods or beverages, and follow the supplement-intake and pre-test restrictions. Group-specific baseline characteristics are reported in Section 3.1.

A feasibility sample of 48 athletes was enrolled and allocated equally among four groups (12/group). Enrollment was not determined by a prospective power calculation. A four-group omnibus sensitivity analysis was performed in Python 3.13.5 using statsmodels 0.14.6. With *N* = 48, two-sided α = 0.05, and 80% power, the design could detect Cohen’s f = 0.50 (approximately η^2^ = 0.20). The study therefore had limited sensitivity for smaller effects, particularly ingredient interactions.

### 2.2. Ethics Approval

The study protocol was reviewed and approved by the Ethics Committee of Beijing Sport University on 26 May 2026 (approval no. 2026052H). All procedures complied with the Declaration of Helsinki [35]. All participants provided written informed consent and could withdraw without penalty. Reporting was prepared with reference to the CONSORT 2025 statement [36].

The trial was retrospectively registered at ClinicalTrials.gov on 30 June 2026 (NCT07690085; Unique Protocol ID: 2026252H). Prospective registration was missed because the investigators initially classified the project as an exercise-performance and sports-nutrition intervention in healthy collegiate athletes rather than an interventional trial requiring prospective registration. After the registration requirement was re-evaluated, the record was completed using the ethics-approved protocol and the procedures actually implemented. The retrospective registry record is reported transparently and is not presented as evidence of prospective outcome specification.

### 2.3. Study Design and Outcomes

This randomized, blinded, placebo-controlled, parallel-group trial compared CTR, CAF, RHO, and RHO + CAF during a four-week standardized Jive-specific training intervention.

**Figure 1:**
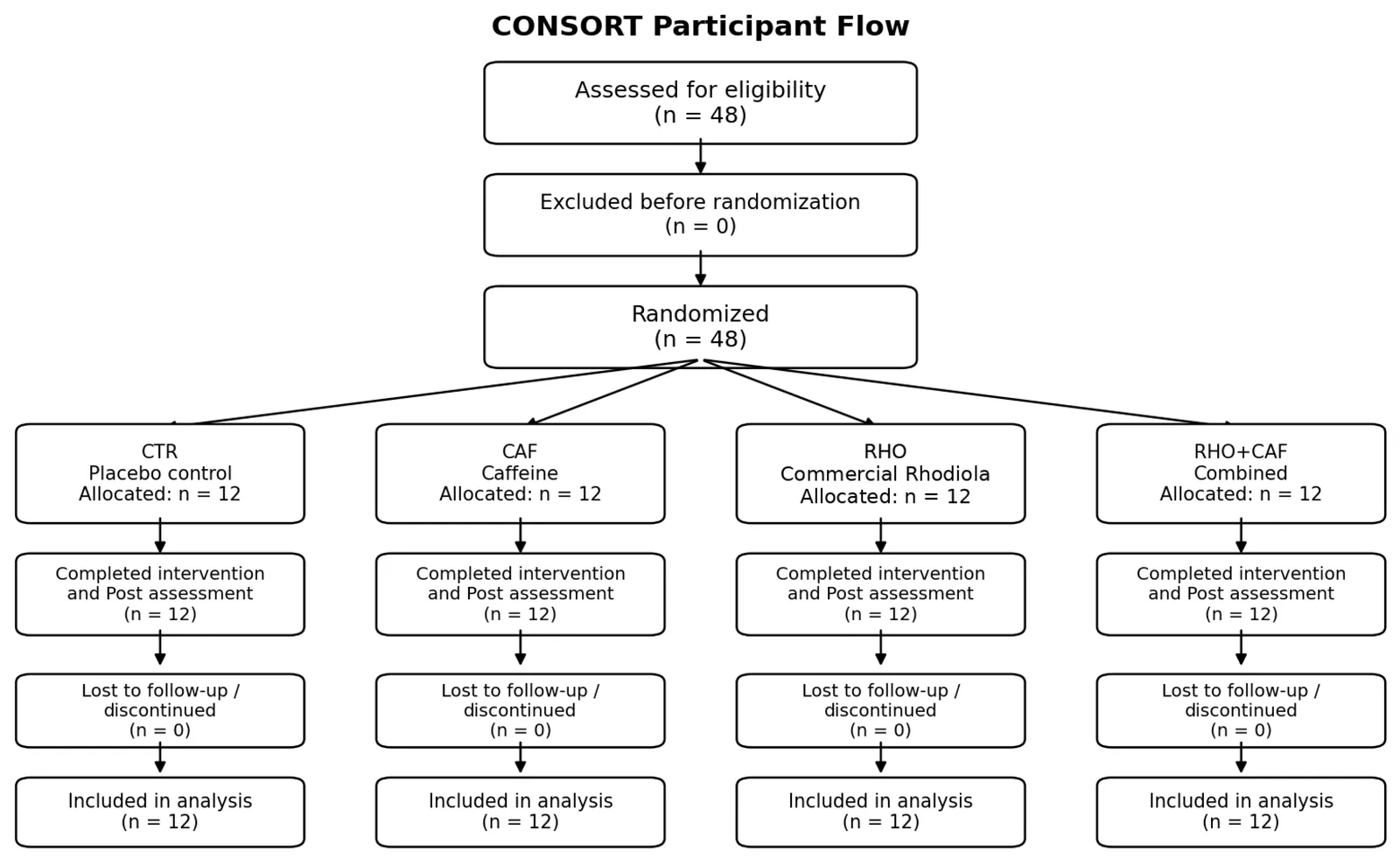
CONSORT participant flow. Forty-eight athletes were assessed for eligibility, none were excluded, and 48 were randomized in a 1:1:1:1 ratio to CTR, CAF, RHO, or RHO + CAF (*n* = 12/group). No participant discontinued or was lost to follow-up, and all 48 were analyzed in their assigned groups.

After the Pre assessment, participants were randomly allocated by drawing lots in a 1:1:1:1 ratio to CTR, CAF, RHO, or RHO + CAF (*n* = 12/group). An independent researcher recorded the draw, retained the allocation-code key, and prepared participant-specific coded supplement packages. The participant-level allocation record and supplement-code log are retained under the study’s ethical and privacy safeguards and are not publicly disclosed. Post assessment followed the four-week intervention. Testing time, sequence, venue, equipment settings, musical rhythm, instructions, and scoring procedures were standardized across Pre and Post. Participant enrollment, randomization, follow-up, and analysis are summarized in Figure 1.

The registry lists four co-primary outcomes representing distinct Jive-specific constructs: kick-extension completion, post-kick return control, trunk stability, and integrated repeated-kick success. The 30 s valid-kick success rate was treated as the principal integrated outcome for interpretation because it summarizes the proportion of 32 prescribed kicks that met all technical criteria. The four primary outcomes were analyzed as one multiplicity-controlled family. Registry-listed secondary outcomes were valid-kick count, YBT composite balance, bird-dog hold time, split-lunge squat valid count, step-up/box step valid count, 45 s high-knee rhythm compliance, 505 change-of-direction time, Illinois agility time, hexagon-jump time, and session-RPE. Because success rate is an exact transformation of valid-kick count, the count was retained as its numerator and was not subjected to a duplicate inferential test.

The investigator responsible for supplement distribution received only participant-specific coded packages and did not have access to the allocation-code key. Participants, the supplement-distribution investigator, outcome assessors, and the statistical analyst remained blinded to treatment allocation until the primary analysis was completed, after which the code was released. All groups completed the same Jive-specific training program; supplementation was the planned between-group difference. No formal allocation-guess assessment was administered; therefore, the manuscript reports the masking procedure but does not claim independently demonstrated blinding success.

### 2.4. Supplement Preparation and Supplementation Protocol

The *Rhodiola rosea* product used in this study was Tongrentang Brand Rhodiola Capsule (Beijing Tongrentang Health Pharmaceutical Co., Ltd., Beijing, China; Chinese health-food registration no. G20100243; batch no. 2025202010). Each bottle contained 120 capsules, each containing 0.4 g of product (net content 48 g). Participants assigned to RHO-containing conditions consumed three capsules twice daily, 30 min before breakfast and dinner, for four weeks, corresponding to 2.4 g·day^−1^ of product mass. The daily capsule amount matched the manufacturer-labeled use of three capsules twice daily. The product label declared salidroside at 0.4 g per 100 g of product; at the administered dose, this corresponds to a label-derived nominal salidroside intake of approximately 9.6 mg·day^−1^. The declared constituent content was not independently verified, and the label did not specify the extraction ratio or standardized-extract status. Product photographs and English-translated label details are provided in Supplementary Product Documentation S1. Anhydrous CAF (Nutricost, Vineyard, UT, USA) was administered at 3 mg·kg^−1^ body mass as a single acute dose 30 min before Post testing. Individual CAF doses were calculated from each participant’s recorded body mass and prepared in participant-specific coded capsules by the independent researcher who retained the allocation-code key. Active and corresponding placebo capsules were matched in external appearance and administration schedule. The investigator responsible for distribution dispensed only the coded packages and did not have access to the allocation-code key. The 30 min interval was fixed in the study protocol to standardize test-day administration across active and placebo conditions. Because this interval is shorter than the commonly used 45–60 min window for caffeine capsules, the findings are interpreted specifically within the tested 30 min protocol and do not assume peak plasma caffeine exposure [11].

Pre assessment was completed before active supplementation. During the four-week phase, RHO and RHO + CAF received RHO, whereas CTR and CAF received the chronic matched placebo. At Post, CAF and RHO + CAF received body-mass-adjusted CAF 30 min before testing, whereas CTR and RHO received the acute matched placebo. The investigator responsible for supplement distribution dispensed the scheduled coded active and placebo capsules without access to the allocation-code key. All 48 participants completed the four-week intervention and Post assessment, and no participant discontinued. Participants maintained their habitual diets and avoided additional caffeine-containing products and sport nutrition supplements. A group-specific numerical pill-adherence percentage was not retained; completion of follow-up and the 100% training-attendance rate are therefore reported separately from dose adherence.

### 2.5. Training Plan

During the four-week intervention, all participants completed the same standardized Jive-specific DanceSport training program on Monday, Tuesday, Thursday, and Saturday. Each session lasted 75 min. Training frequency, duration, content, intensity, and recovery intervals were standardized across groups. Attendance was recorded for every session, and all participants attended all scheduled sessions, corresponding to an overall training attendance rate of 100%. All 48 participants completed the four-week intervention and Post assessment. The weekly plan is shown in Table 1.

Pre and Post assessments were scheduled outside the high-intensity training sessions. Participants avoided high-intensity routine practice, lower-limb explosive training, and heavy resistance training for 24 h before each assessment. Testing time and recovery conditions were standardized across Pre and Post.

### 2.6. Daily Diet and Lifestyle Control

Throughout the experiment, all participants were required to maintain their habitual diet and daily lifestyle and to avoid intentional changes in total energy intake or macronutrient distribution. Standardizing caffeine and stimulant exposure is important when interpreting exercise-performance responses [11,12]. Evidence from endurance and team-sport settings further supports controlling acute caffeine exposure around performance testing [13,20]. Therefore, participants were prohibited from using additional sport nutrition supplements, ergogenic aids, or other stimulants affecting performance. Participants were also required to restrict caffeine-containing foods and beverages and to avoid coffee, strong tea, energy drinks, cola, and other caffeine sources within 48 h before each formal test.

Diet, sleep, and non-study activity were standardized by instruction and were not analyzed as quantitative efficacy outcomes.

To ensure consistent Pre/Post conditions, all tests were performed between 15:00 and 17:00, and each participant’s Pre and Post testing times differed by no more than ±1 h. Within 24 h before each test, participants were required to avoid strenuous exercise, alcohol intake, and staying up late, and to reproduce the previous test-day evening meal and pre-test dietary pattern. On the test day, participants maintained normal hydration but avoided excessive fluid or large meal intake shortly before testing.

### 2.7. Testing Procedures and Standardization

Baseline assessment (Pre) and post-intervention assessment (Post) followed the same standardized procedure. Participants completed a familiarization session before baseline testing to reduce learning effects. In this manuscript, perceived fatigue refers to task-specific cumulative load during repeated Jive actions rather than systemic fatigue.

To reduce residual fatigue and task-order bias, all participants completed the tests in a fixed order, with standardized passive recovery intervals between major testing tasks. The sequence was as follows: (1) standardized warm-up; (2) kick-extension angle test; (3) post-kick leg-retraction angle test; (4) trunk anteroposterior sway amplitude test; (5) 30 s specific kick task; (6) YBT; (7) bird-dog core stability test; (8) split-lunge squat and step-up/box step tests; (9) high-knee running-in-place test; (10) 505 change-of-direction test; (11) Illinois agility run; (12) hexagon jump test; and (13) session-RPE recording.

All formal tests followed the same fixed order, standardized warm-up, footwear requirements, verbal instructions, equipment settings, and recovery intervals. Two synchronized smartphone cameras (realme GT8 Pro; 2160 × 3840 pixels; 60 frames·s^−1^, realme, Shenzhen, China) recorded the Jive trials. The standardized lateral-view video was imported into Kinovea software for frame-by-frame two-dimensional angle analysis, and the second view was used to verify movement timing, technique criteria, and assessor scoring. No calibrated three-dimensional reconstruction was performed. The same camera placement and recording configuration were used at Pre and Post.

A 3 min standardized passive recovery interval was used between major testing tasks; for continuous or high-intensity tasks, recovery was extended to 5 min to allow participants to complete subsequent tests in a relatively stable condition. The test venue, equipment configuration, testing order, musical rhythm, operating procedure, and testing staff were kept consistent across Pre and Post. Personnel responsible for data collection, recording, and outcome determination remained blinded to treatment allocation, and the statistical analyst used coded group labels until completion of the primary analysis.

### 2.8. Sport-Specific Kick Kinematics: Extension and Retraction Angles

Kick-extension and post-kick leg-retraction angles were derived from synchronized recordings obtained with two realme GT8 Pro smartphones (2160 × 3840 pixels; 60 frames·s^−1^). The standardized lateral-view video was analyzed frame by frame in Kinovea using hip, knee, ankle, toe, trunk, and pelvic landmarks; the second view verified movement events and scoring decisions. Each kinematic outcome was averaged across three valid trials.

After the standardized warm-up, participants performed a low forward Jive kick in standardized Latin dance training shoes. A valid trial required a stable support leg, maximum knee-extension angle > 175°, pointed instep, toe clearance > 5 cm, and no toe drag, marked support-foot displacement, rhythm error, or excessive trunk compensation. Angles ≥ 178° were classified as excellent for descriptive interpretation; values closer to 180° indicated more complete extension. Three valid trials were separated by 60–90 s of passive recovery.

Kick-extension angle was defined by the hip–knee–ankle angle at maximal extension. Post-kick leg-retraction angle was the maximum return angle reached after kick completion and before the next movement; ≥50° was acceptable and ≥60° represented a higher technical level. The mean of three valid trials was used for each analysis.

**Figure 2:**
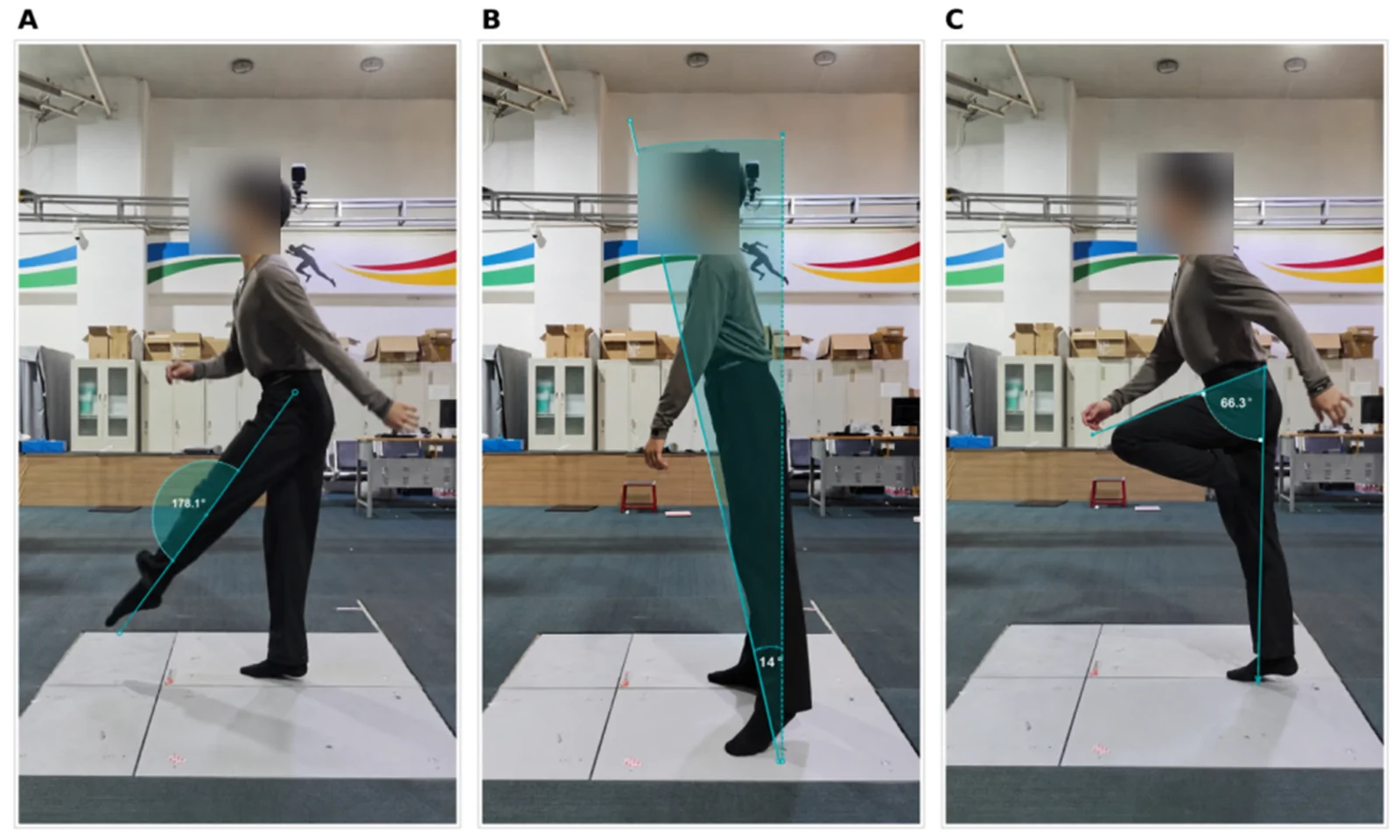
Representative high-definition video frames used for the registry-listed Jive-specific kinematic assessments: (**A**) kick-extension angle; (**B**) trunk anteroposterior sway amplitude; and (**C**) post-kick leg-retraction angle. Angles were calculated in Kinovea from the standardized lateral-view recording; the second synchronized view was used for verification. Knee extension > 175° was valid and ≥178° was excellent; values closer to 180° indicated more complete extension. The displayed values illustrate the measurement procedure and are not group-level results. Faces were obscured. Recordings were acquired using two realme GT8 Pro smartphones at 2160 × 3840 pixels and 60 frames·s^−1^.

Representative field images of the three registry-listed kinematic assessments are shown in Figure 2.

### 2.9. Thirty-Second Repeated-Kick Test

This test assessed whether athletes could maintain technique and rhythm across eight eight-counts (32 prescribed kicks) in 30 s. The trial was recorded with the same two-camera setup; the standardized lateral view was reviewed frame by frame in Kinovea, and the second view was used for cross-checking.

A kick was classified as valid only when all criteria were satisfied: maximum knee-extension angle > 175°; toe clearance > 5 cm with the instep pointed; post-kick leg-retraction angle ≥ 50°; trunk anteroposterior sway ≤ 30°; and no clear rhythm interruption, toe drag, marked support-foot displacement, or kick-direction deviation. Angles ≥ 178° were classified as excellent for descriptive interpretation, and values closer to 180° indicated better technical completion; this classification did not change the binary valid-kick score.

Two assessors blinded to allocation independently scored all 32 kicks from the synchronized recordings; disagreements were resolved by a third assessor. Valid-kick count ranged from 0 to 32. The registry-listed primary 30 s valid-kick success rate was calculated for each participant as valid-kick count/32 × 100. Because the percentage is an exact linear transformation of the count, the count was retained as the registry-listed secondary numerator without a separate inferential test.

### 2.10. Movement Stability Assessment: Trunk Sway, YBT Balance, and Core Stability Tests

Trunk anteroposterior sway amplitude, YBT composite balance, and bird-dog hold time were used to characterize complementary aspects of Jive-related movement stability and core control.

Trunk anteroposterior sway amplitude was quantified from the synchronized recordings. The standardized lateral-view video was analyzed frame by frame in Kinovea using trunk and pelvic landmarks, and the second view was used for verification. Lower values indicated less anteroposterior sway.

The YBT was performed using a dynamic and static balance testing and training system. Participants stood on one leg at the center of the testing system and performed a maximal reach with the contralateral limb in the anterior, posteromedial, and posterolateral directions. The support foot was required to remain stable, with no obvious body sway or compensatory force. After touching the target point with the reaching foot, the participant returned to the starting position. The system recorded reach distance in each direction and calculated a composite balance score according to lower-limb length. This variable reflected single-leg support, weight transfer, and dynamic balance control in Jive. YBT/SEBT-type tests have substantial reliability and validity evidence for dynamic postural control, lower-limb functional assessment, and injury-risk screening [37,38,39].

Core stability was assessed using the bird-dog hold test. Participants maintained the standardized contralateral arm-and-leg extension with a neutral pelvis and without trunk rotation, lumbar collapse, or support-point movement. Effective hold time was recorded in seconds, and the test ended at the first loss of the standardized posture.

Testing commands, sequence, and recovery intervals were standardized. Trunk sway was averaged across three valid trials. YBT and bird-dog testing followed familiarization practice; trials with support-foot displacement, imbalance, movement-path error, or obvious compensation were repeated. Representative sequences are provided in Supplementary Figure S1.

### 2.11. Supporting Physical-Capacity Tests and Perceived Internal Load Assessment

Repeated kick-and-retraction is an indispensable technical component of Jive, and the 30 s repeated-kick task directly assessed the sustained quality of this fundamental dance action. The additional field tests were used to quantify the physical capacities that support high-quality and repeatable Jive execution rather than to substitute for direct dance-technique assessment. Specifically, they assessed lower-limb force production, rhythm maintenance, stop–restart ability, multidirectional agility, and jump–landing control. Split-lunge squat and step-up/box step were tested separately, with 4 sets of 12 repetitions (48 repetitions per task). For both tasks, a repetition was valid only when the prescribed start-to-finish movement was completed in rhythm, the trunk remained stable, the knee tracked in line with the foot, the required movement range was completed, and no balance loss or external support occurred. High-knee running in place was evaluated using a 45 s rhythm-compliance rate. A beat was compliant when the alternating knee lift reached the standardized target used during familiarization, the foot returned to the prescribed landing position, arm action remained coordinated, and no beat was missed or delayed. The 505 change-of-direction test assessed deceleration, turning, and re-acceleration ability, whereas the Illinois agility run assessed linear acceleration, slalom movement, and multidirectional change-of-direction ability [40,41,42]. Completion time was recorded for both tests. The hexagon jump test assessed multidirectional repeated jumping, landing buffering, and jump coordination; the time required to complete the prescribed route was recorded. Timed tests retained the best performance, whereas count and compliance-rate tests recorded valid completion within the prescribed task. These supporting tests characterize the strength, control, rhythm, braking, re-acceleration, and coordination capacities that form the physical foundation for better Jive technique [43,44,45]. Representative testing sequences for the split-lunge squat, step-up/box step, and high-knee running-in-place tests are provided in Supplementary Figure S2. The 505 change-of-direction and Illinois agility-run sequences are provided in Supplementary Figure S3.

Perceived internal load was recorded using a 10-point RPE scale, where 1 indicated almost no exertion and 10 indicated maximal exertion [46,47]. After all testing tasks, participants rested quietly for 1–2 min and independently reported their overall perceived exertion. This registry-listed variable is retained as session-RPE throughout the manuscript for consistency with the trial record.

### 2.12. Statistical Analysis

Analyses were conducted using SPSS Statistics 27.0 and GraphPad Prism 10. Continuous variables are reported as mean ± SD. For each registry-listed outcome, Post was analyzed by ANCOVA with Group as the fixed factor and the corresponding Pre value as covariate. Residual distributions, variance patterns, influential observations, and homogeneity of regression slopes were examined. When the Group × Pre term indicated non-parallel slopes, it was retained and adjusted group estimates were calculated at the overall Pre mean. Tests were two-sided with α = 0.05. Adjusted means and mean differences are reported with 95% CIs, and effect magnitude is summarized using partial η^2^. Model-specific diagnostics are reported in Supplementary Data S1. The Group × Pre interaction was retained for trunk anteroposterior sway and 30 s valid-kick success rate; adjusted means and contrasts for these models were evaluated at the overall Pre mean. HC3-robust sensitivity analyses were used to assess whether the conclusions were sensitive to residual variance departures.

Holm’s procedure controlled family-wise error across the four registry-listed primary ANCOVA tests. The 30 s valid-kick success rate was the principal integrated outcome for interpretation, while all four outcomes remained within the same multiplicity-controlled family. Benjamini–Hochberg false-discovery-rate correction was applied across the nine non-redundant secondary ANCOVA tests. Following a significant ANCOVA, Bonferroni-adjusted pairwise comparisons were reported with adjusted mean differences and 95% CIs. A supplementary 2 × 2 factorial analysis coded CAF and RHO as two binary between-participant factors and analyzed Pre–Post change. In this two-timepoint formulation, the CAF × RHO interaction in change tests the same non-additivity addressed by a CAF × RHO × Time interaction. Interaction results were used to test departure from additivity and were not inferred from pairwise group differences. Formal intervention-effect inference was based on baseline-adjusted ANCOVA and multiplicity-controlled contrasts, with complete estimates and diagnostics provided in Supplementary Data S1.

## 3. Results

### 3.1. Baseline Characteristics

Forty-eight athletes were assessed for eligibility; none were excluded before randomization. All 48 were randomized equally to CTR, CAF, RHO, and RHO + CAF (*n* = 12/group), completed the four-week intervention and Post assessment, and were analyzed in their assigned groups. No participant was lost to follow-up or discontinued. Overall training attendance was 100%. Adverse events were not systematically quantified; consequently, the absence of discontinuation should not be interpreted as evidence of supplement safety. Group-specific baseline characteristics are reported in Table 2; registry-listed Pre values and baseline-adjusted results are presented in Table 3, Section 3.2, Section 3.3, Section 3.4 and Section 3.5, and Supplementary Data S1.

**Figure 3:**
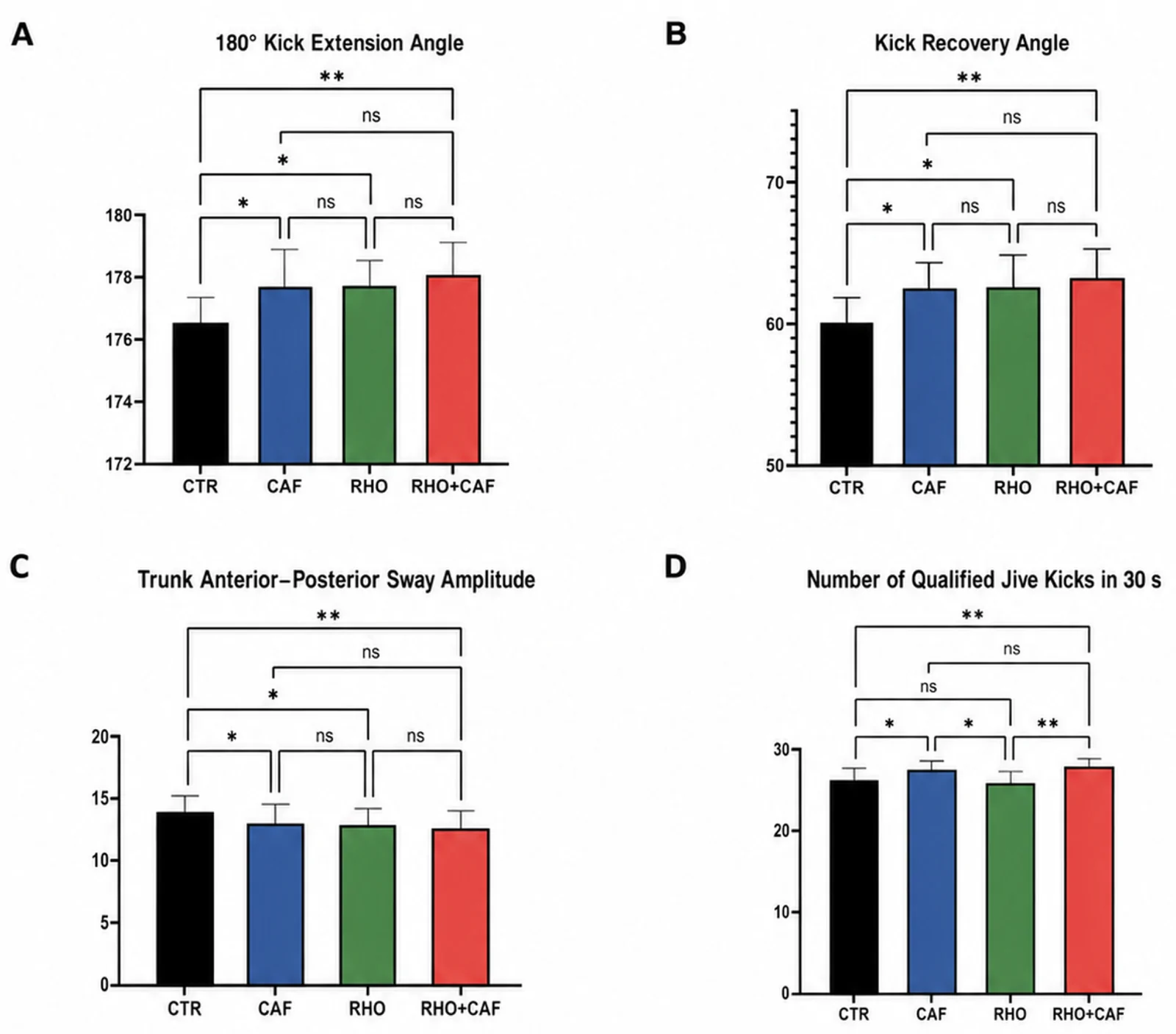
Unadjusted Post mean ± SD plots for sport-specific outcomes: (**A**) kick-extension angle; (**B**) post-kick leg-retraction angle; (**C**) trunk anteroposterior sway amplitude; and (**D**) 30 s valid-kick count. The registered 30 s valid-kick success rate was calculated at the participant level as valid-kick count/32 × 100 and is reported in Section 3.2 and Supplementary Data S1. In-figure Tukey annotations describe unadjusted Post comparisons; primary intervention-effect inference is based on the baseline-adjusted ANCOVA results. Annotation key for the descriptive Post-only Tukey comparisons: ns, *p* ≥ 0.05; * *p* < 0.05; ** *p* < 0.01.

**Figure 4:**
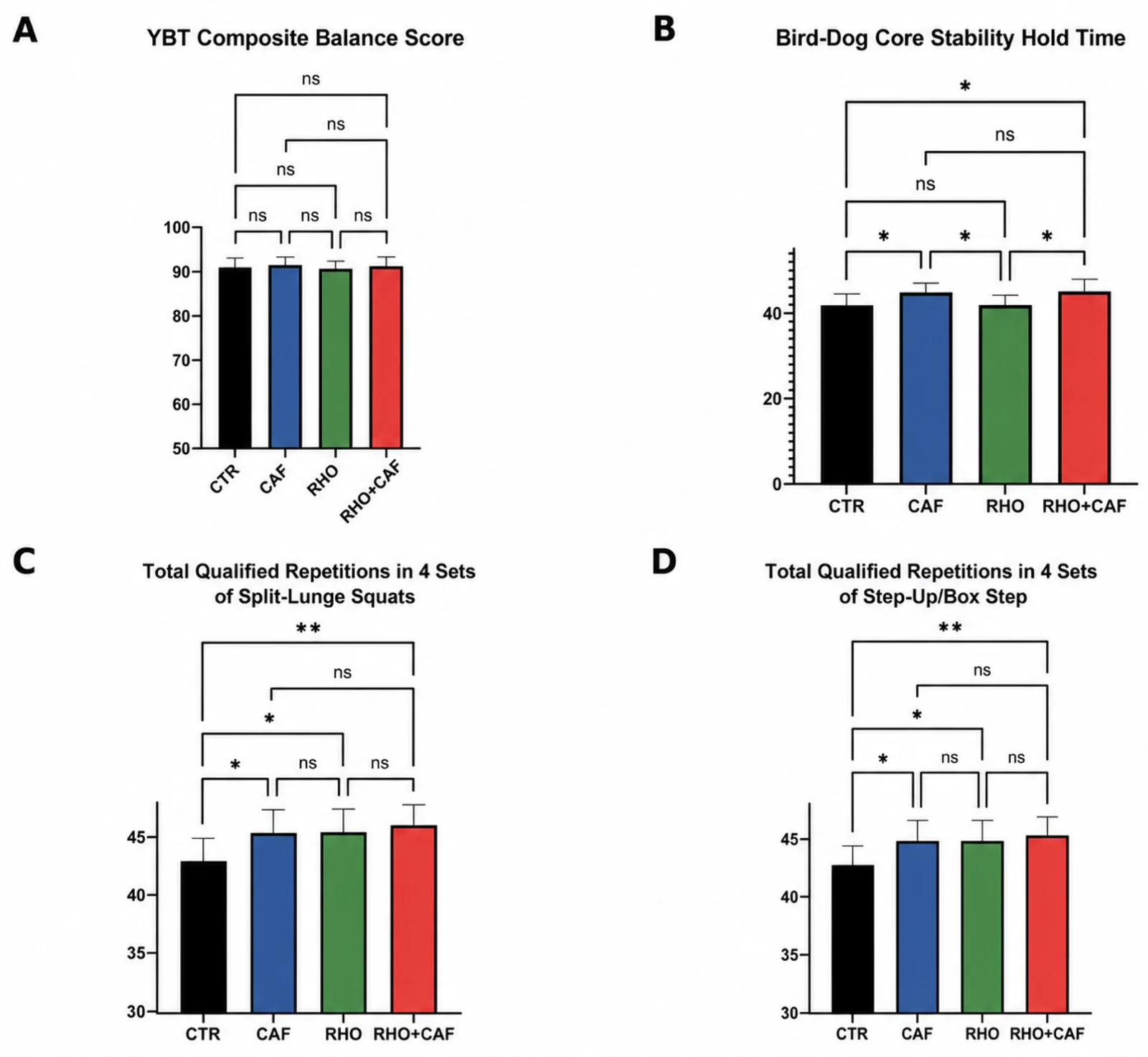
Unadjusted Post mean ± SD plots for secondary stability and specific-strength outcomes: (**A**) YBT composite balance; (**B**) bird-dog hold time; (**C**) split-lunge squat valid count; and (**D**) step-up/box step valid count. In-figure annotations refer to unadjusted Post comparisons; baseline-adjusted results are reported in Section 3.3 and Supplementary Data S1. Annotation key for the descriptive Post-only Tukey comparisons: ns, *p* ≥ 0.05; * *p* < 0.05; ** *p* < 0.01.

**Figure 5:**
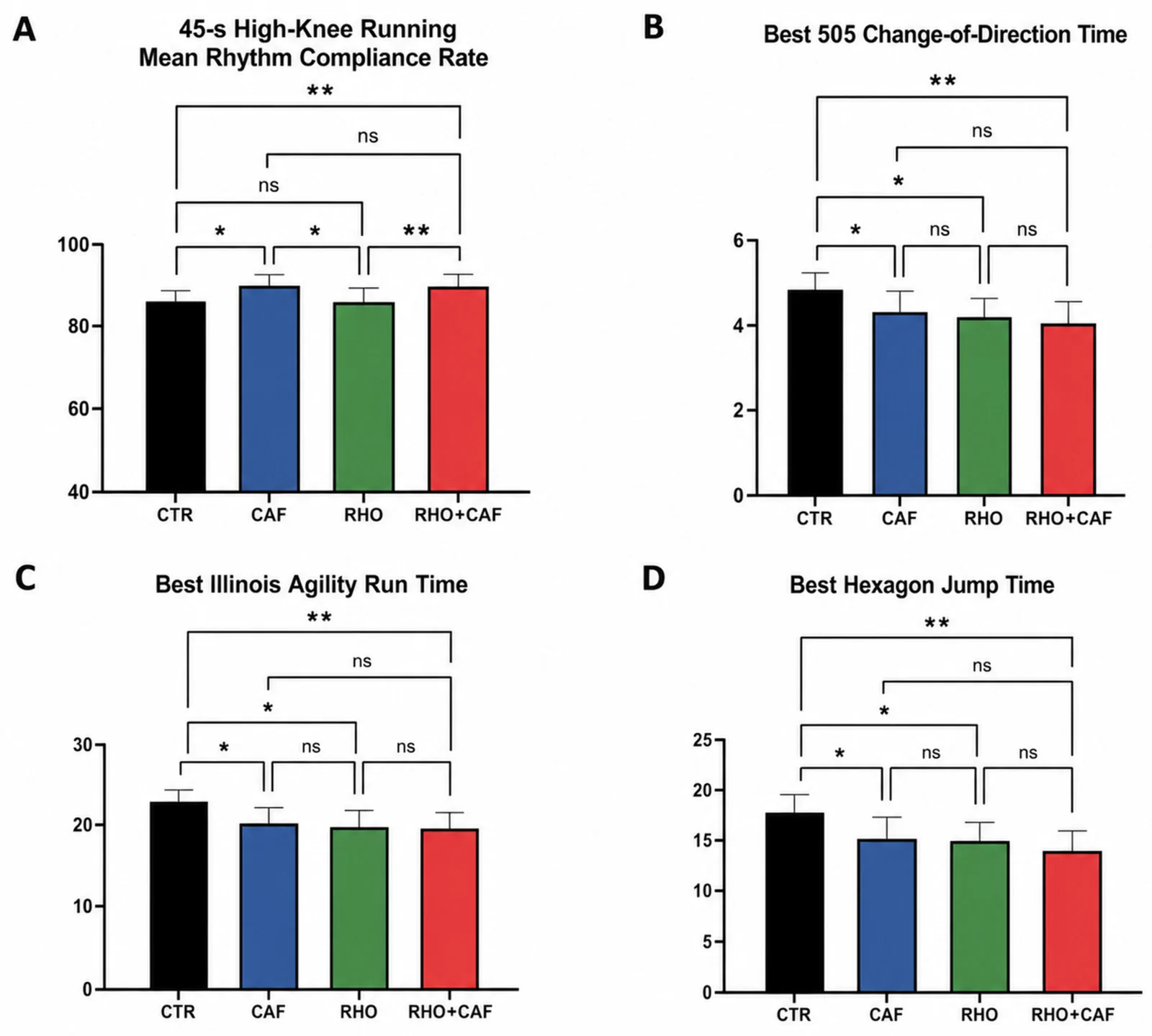
Unadjusted Post mean ± SD plots for secondary rhythm, agility, and multidirectional outcomes: (**A**) 45 s high-knee rhythm compliance; (**B**) 505 change-of-direction time; (**C**) Illinois agility time; and (**D**) hexagon-jump time. In-figure annotations refer to unadjusted Post comparisons; baseline-adjusted results are reported in Section 3.4 and Supplementary Data S1. Annotation key for the descriptive Post-only Tukey comparisons: ns, *p* ≥ 0.05; * *p* < 0.05; ** *p* < 0.01.

**Figure 6:**
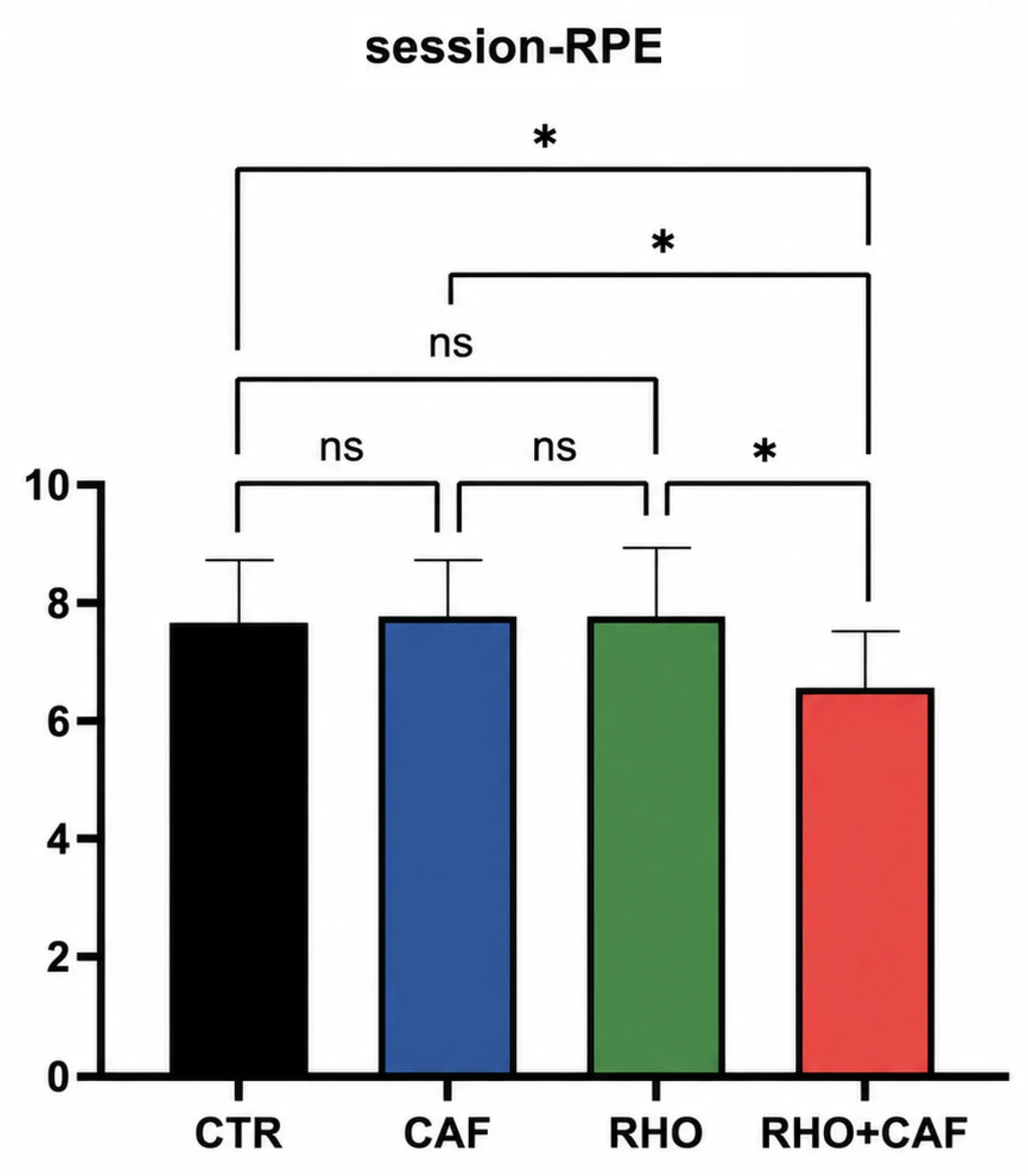
Unadjusted Post mean ± SD plot for session-RPE. In-figure annotations refer to unadjusted Post comparisons; the baseline-adjusted result is reported in Section 3.5 and Supplementary Data S1. Annotation key for the descriptive Post-only Tukey comparisons: ns, *p* ≥ 0.05; * *p* < 0.05.

Primary interpretation was based on ANCOVA using the corresponding Pre value as covariate. All four registry-listed primary-outcome omnibus tests remained significant after Holm correction. Among the nine non-redundant secondary outcomes, all except YBT met the Benjamini–Hochberg criterion. Figure 3, Figure 4, Figure 5 and Figure 6 provide unadjusted Post summaries; the principal conclusions follow the baseline-adjusted analyses. The Group × Pre interaction was retained for trunk anteroposterior sway (*p* = 0.0274) and 30 s valid-kick success rate (*p* = 0.00816). HC3-robust sensitivity analyses yielded the same omnibus significance conclusions; full diagnostics are reported in Supplementary Data S1.

### 3.2. Registry-Listed Primary Outcomes

For kick-extension angle, adjusted Post means (95% CIs) were 176.57° (175.97–177.16) in CTR, 177.68° (177.08–178.27) in CAF, 177.73° (177.14–178.33) in RHO, and 178.03° (177.43–178.62) in RHO + CAF. The group effect was significant (raw *p* = 0.006182, Holm-adjusted *p* = 0.006182, partial η^2^ = 0.248). Significant Bonferroni-adjusted contrasts (mean difference, 95% CI) were observed for RHO versus CTR (+1.17°, 0.01–2.32, *p* = 0.046) and RHO + CAF versus CTR (+1.46°, 0.30–2.61, *p* = 0.0066); CAF versus CTR was not significant (Figure 3A).

For post-kick leg-retraction angle, adjusted Post means (95% CIs) were 60.06° (58.93–61.19), 62.35° (61.22–63.48), 62.49° (61.36–63.62), and 63.03° (61.90–64.16) in CTR, CAF, RHO, and RHO + CAF, respectively. The group effect was significant (raw *p* = 0.002701, Holm-adjusted *p* = 0.005401, partial η^2^ = 0.278). Relative to CTR, adjusted differences (95% CIs) were +2.29° for CAF (0.10–4.49, *p* = 0.036), +2.43° for RHO (0.24–4.63, *p* = 0.022), and +2.97° for RHO + CAF (0.78–5.17, *p* = 0.0031) (Figure 3B).

For trunk anteroposterior sway amplitude, adjusted Post means (95% CIs) were 14.02° (13.49–14.56) in CTR, 12.81° (12.28–13.35) in CAF, 12.77° (12.23–13.31) in RHO, and 12.56° (12.02–13.10) in RHO + CAF. The group effect was significant (raw *p* = 0.001432, Holm-adjusted *p* = 0.004295, partial η^2^ = 0.318). Relative to CTR, adjusted differences (95% CIs) were −1.21° for CAF (−2.25 to −0.16, *p* = 0.0157), −1.25° for RHO (−2.30 to −0.20, *p* = 0.0116), and −1.46° for RHO + CAF (−2.51 to −0.42, *p* = 0.0022) (Figure 3C).

For the registry-listed 30 s valid-kick success rate, adjusted Post means (95% CIs) were 84.66% (81.82–87.50) in CTR, 90.69% (87.85–93.53) in CAF, 84.43% (81.59–87.27) in RHO, and 92.31% (89.47–95.15) in RHO + CAF. The group effect was significant (raw *p* = 0.000192, Holm-adjusted *p* = 0.000768, partial η^2^ = 0.386). Significant Bonferroni-adjusted differences (mean difference, 95% CI) were CAF versus CTR (+6.03 percentage points, 0.51–11.55, adjusted *p* = 0.0254), RHO + CAF versus CTR (+7.64, 2.13–13.16, adjusted *p* = 0.0025), CAF versus RHO (+6.26, 0.74–11.78, adjusted *p* = 0.0186), and RHO + CAF versus RHO (+7.88, 2.36–13.39, adjusted *p* = 0.0018). Corresponding raw Post counts were 27.08 ± 1.98, 29.00 ± 1.48, 27.00 ± 1.91, and 29.50 ± 1.45 (Figure 3D).

### 3.3. Registry-Listed Secondary Stability and Specific-Strength Outcomes

YBT composite balance did not differ among groups after baseline adjustment (raw *p* = 0.750784, Benjamini–Hochberg q = 0.750784, partial η^2^ = 0.027) (Figure 4A).

Bird-dog core stability hold time differed among groups (raw *p* = 0.001161, q = 0.003734, partial η^2^ = 0.307). Adjusted Post means were 42.07, 44.97, 42.10, and 45.26 s for CTR, CAF, RHO, and RHO + CAF. Bonferroni-adjusted contrasts showed higher bird-dog hold times in CAF than CTR (*p* = 0.0313) and RHO (*p* = 0.0335), and in RHO + CAF than CTR (*p* = 0.0138) and RHO (*p* = 0.0148); CAF and RHO + CAF did not differ significantly (Figure 4B).

Split-lunge squat valid count differed among groups (raw *p* = 0.002490, q = 0.003734, partial η^2^ = 0.281). Adjusted Post means were 43.08, 45.24, 45.67, and 46.08 repetitions. Bonferroni-adjusted contrasts showed higher counts in RHO (*p* = 0.0138) and RHO + CAF (*p* = 0.0031) than CTR. The CAF–CTR contrast did not remain significant after adjustment (*p* = 0.0591), and no contrasts among the active groups were significant (Figure 4C).

Step-up/box step valid count differed among groups (raw *p* = 0.003138, q = 0.004035, partial η^2^ = 0.273). Adjusted Post means were 43.02, 45.33, 45.52, and 45.64 repetitions. Bonferroni-adjusted contrasts showed higher counts in CAF (*p* = 0.0235), RHO (*p* = 0.0121), and RHO + CAF (*p* = 0.0077) than CTR; no contrasts among the active groups were significant (Figure 4D).

### 3.4. Registry-Listed Secondary Rhythm, Agility, and Multidirectional Outcomes

Forty-five-second high-knee rhythm compliance differed among groups (raw *p* = 0.000870, q = 0.003734, partial η^2^ = 0.317). Adjusted Post means were 86.07%, 90.98%, 86.01%, and 92.15%. Bonferroni-adjusted contrasts showed higher compliance in CAF than CTR (*p* = 0.0489) and RHO (*p* = 0.0446), and in RHO + CAF than CTR (*p* = 0.0079) and RHO (*p* = 0.0072); CAF and RHO + CAF did not differ significantly (Figure 5A).

The 505 change-of-direction time differed among groups (raw *p* = 0.001557, q = 0.003734, partial η^2^ = 0.297). Adjusted Post means were 4.89, 4.32, 4.22, and 4.06 s. Bonferroni-adjusted contrasts showed faster times in RHO (*p* = 0.0138) and RHO + CAF (*p* = 0.0015) than CTR. The CAF–CTR contrast did not remain significant after adjustment (*p* = 0.0501), and no contrasts among the active groups were significant (Figure 5B).

Illinois agility time differed among groups (raw *p* = 0.002275, q = 0.003734, partial η^2^ = 0.284). Adjusted Post means were 23.28, 19.99, 19.93, and 19.25 s. Bonferroni-adjusted contrasts showed faster times in CAF (*p* = 0.0217), RHO (*p* = 0.0191), and RHO + CAF (*p* = 0.0031) than CTR; no contrasts among the active groups were significant (Figure 5C).

Hexagon-jump time differed among groups (raw *p* = 0.002465, q = 0.003734, partial η^2^ = 0.281). Adjusted Post means were 17.98, 15.34, 15.15, and 14.66 s. Bonferroni-adjusted contrasts showed faster times in CAF (*p* = 0.0299), RHO (*p* = 0.0164), and RHO + CAF (*p* = 0.0035) than CTR; no contrasts among the active groups were significant (Figure 5D).

### 3.5. Session-RPE and Ingredient Interaction

Session-RPE differed among groups after baseline adjustment (raw *p* = 0.007321, q = 0.008236, partial η^2^ = 0.241); adjusted Post means were 7.58, 7.72, 7.75, and 6.45 for CTR, CAF, RHO, and RHO + CAF. Bonferroni-adjusted contrasts showed lower session-RPE in RHO + CAF than CTR (*p* = 0.0477), CAF (*p* = 0.0186), and RHO (*p* = 0.0198); no other pairwise contrasts were significant (Figure 6). In the supplementary 2 × 2 analysis, the CAF × RHO interaction for valid-kick success rate was 2.08 percentage points (95% CI −7.65 to 11.81); *p* = 0.668. No CAF × RHO interaction was detected for any registry-listed primary outcome (*p* = 0.2665–0.6682), and the interaction tests did not support departure from additivity.

## 4. Discussion

This randomized trial with retrospective registration compared CAF, RHO, and RHO + CAF during a standardized four-week Jive-specific training program in male collegiate DanceSport athletes. After adjustment for the corresponding Pre value, all four registry-listed primary-outcome tests remained significant after Holm correction, with partial η^2^ values of 0.248–0.386. Eight of nine non-redundant secondary outcomes met the Benjamini–Hochberg criterion, whereas YBT did not. The pattern indicates outcome-specific group differences rather than uniform improvement across all tests. At the adjusted group-mean level, every group exceeded the >175° validity threshold for knee extension, whereas only the RHO + CAF mean reached the ≥178° excellent threshold; this group-level comparison does not imply that every participant met the excellent criterion.

CAF-related differences were concentrated in repeated technical execution, movement control, rhythm, and selected multidirectional tasks. Compared with CTR, CAF produced a 6.03-percentage-point higher adjusted 30 s valid-kick success rate, a 2.29° greater post-kick leg-retraction angle, and a 1.21° lower trunk anteroposterior sway amplitude. CAF also showed favorable pairwise differences in bird-dog core stability, 45 s high-knee rhythm compliance, Illinois agility performance, and hexagon-jump performance. By contrast, the Bonferroni-adjusted CAF–CTR comparison for kick-extension angle did not reach significance, and CAF did not show a significant adjusted advantage over CTR in the 505 test. This pattern supports a task-specific interpretation: under the present protocol, CAF was most clearly associated with maintaining valid repeated kicks, controlling the trunk, completing the retraction phase, and sustaining rhythm-dependent output. Broad reviews support acute ergogenic effects of CAF across multiple exercise domains [11,12]. Meta-analyses report benefits for endurance and muscular strength/endurance [13,14], while strength-and-power effects have also been documented [15]. Low-dose and inter-individual-response evidence indicates that caffeine effects can vary by dose and participant [16,17]. Intermittent-sprint studies likewise show task-dependent responses [18,19]. Team-sport evidence supports similar context dependence [20,21]. Because neural drive, muscle activation, and central-fatigue variables were not measured, the present data describe performance outcomes and do not establish those physiological mechanisms.

RHO produced a different outcome profile. Relative to CTR, RHO showed a 1.17° greater adjusted kick-extension angle, a 2.43° greater post-kick leg-retraction angle, and a 1.25° lower trunk sway amplitude. Favorable adjusted differences were also observed for the 505 change-of-direction test, Illinois agility run, and hexagon jump. However, the adjusted 30 s valid-kick success rate in RHO did not differ from CTR, and RHO did not show pairwise superiority in bird-dog core stability or 45 s high-knee rhythm compliance. Thus, improvements in selected kinematic and multidirectional outcomes did not translate into a higher proportion of fully valid kicks across the complete 30 s sequence. Acute trials have reported variable endurance-related responses to *Rhodiola rosea* [22,23]. Reviews likewise describe heterogeneous findings across fatigue and exercise-performance outcomes [24,25]. A sport-focused systematic review also emphasizes variation across protocols and outcomes [26]. More recent constituent-specific and football studies suggest that responses may depend on preparation and task [27,28]. A recent meta-analysis further supports heterogeneity across endurance outcomes and related biomarkers [29]. The present results therefore support interpretation at the level of the measured Jive components rather than a general anti-fatigue or metabolic effect.

RHO + CAF showed the broadest favorable pattern across the registry-listed outcomes. Compared with CTR, the combined group had a 1.46° greater adjusted kick-extension angle, a 2.97° greater leg-retraction angle, a 1.46° lower trunk sway amplitude, and a 7.64-percentage-point higher valid-kick success rate. The combined group also had the highest adjusted means for both specific-strength tasks and 45 s rhythm compliance, the fastest adjusted times in the 505, Illinois, and hexagon tests, and the lowest adjusted session-RPE. Nevertheless, the combined group was not significantly superior to both single-supplement groups for kick extension, leg retraction, or trunk sway. More importantly, the supplementary 2 × 2 analysis showed no CAF × RHO interaction for valid-kick success rate or the other registry-listed primary outcomes. The combined condition should therefore be described as producing a broader distribution of favorable observed outcomes, not as demonstrating pharmacological synergy or a departure from additivity. Earlier combined-supplement studies examined strength, muscular endurance, and aerobic-performance outcomes [30,31]. More recent work extended this approach to volleyball and soccer settings [32,33].

Repeated kick-and-retraction is a fundamental and indispensable technical element of Jive. The 30 s task standardized this element across 32 prescribed repetitions to quantify sustained technical quality; it was not treated as a generic fitness drill or as a substitute for the complete competitive routine. The 30 s valid-kick success rate therefore provides the clearest integrated interpretation of the Jive-specific findings. Each kick had to satisfy all criteria: knee-extension angle >175°, toe clearance >5 cm with the instep pointed, post-kick leg retraction ≥50°, trunk anteroposterior sway ≤30°, and uninterrupted rhythmic execution without toe drag, marked support-foot displacement, or kick-direction deviation. Angles ≥178° were classified as excellent for descriptive interpretation without changing the binary valid-kick score. Success rate was calculated as valid-kick count/32 × 100, and the count was not tested again as a duplicate endpoint. CAF and RHO + CAF showed higher adjusted success rates than CTR and RHO, whereas RHO did not differ from CTR. Thus, the CAF-containing conditions were associated with a greater proportion of complete valid repetitions across the 30 s task.

The absence of a group difference in YBT, despite significant differences in trunk sway, bird-dog performance, rhythm, and several agility measures, further demonstrates that generic balance and Jive-specific control are not interchangeable constructs [37,38,39]. YBT evaluates standardized single-leg reach performance, whereas repeated Jive kicking requires rapid alternation between support, propulsion, retraction, trunk stabilization, and musical timing. The 505 and Illinois tests quantify distinct aspects of braking, re-acceleration, directional transition, and multidirectional agility [40,41,42]. The hexagon jump additionally reflects repeated multidirectional jumping and landing control [43,44,45]. The 30 s repeated-kick task directly evaluates an indispensable Jive technical action; the remaining tests characterize the underlying physical capacities that help athletes execute that action with greater quality, control, and repeatability. A multi-indicator framework is therefore appropriate for DanceSport when direct Jive technical outcomes remain the primary basis for interpretation and supporting tests are not treated as substitutes for dance performance.

The statistical framework used the corresponding Pre value to estimate adjusted group differences at Post. Holm correction controlled the primary-outcome family, Benjamini–Hochberg correction addressed multiplicity across non-redundant secondary outcomes, and Bonferroni-adjusted contrasts identified specific between-group differences. Adjusted means, mean differences, 95% CIs, and partial η^2^ provide information beyond isolated *p* values. Applied interpretation should remain specific to the tested protocol: CAF at 3 mg·kg^−1^ body mass 30 min before Post testing, RHO at 2.4 g·day^−1^ for four weeks, and standardized Jive training.

This study has several limitations. First, enrollment was feasibility-based, and the modest sample limited sensitivity for small effects and ingredient interactions. Second, the cohort comprised trained male collegiate athletes aged 18–23 years; women were not enrolled, and the findings cannot address sex-related response heterogeneity or be generalized directly to female dancers, other age groups, or elite international competitors [48]. Third, the four-week intervention does not establish longer-term efficacy or safety. Adverse events were not systematically quantified, so the absence of withdrawal cannot be interpreted as evidence that the supplementation protocol was free of adverse effects. Fourth, two synchronized realme GT8 Pro smartphones recorded at 2160 × 3840 pixels and 60 frames·s^−1^, but Kinovea analysis remained two-dimensional and could not quantify out-of-plane rotation or provide calibrated three-dimensional kinematics. Fifth, biochemical, electromyographic, force-platform, and other mechanistic measurements were not collected; physiological explanations therefore remain outside the evidence generated by this study. Sixth, the label-declared salidroside content (0.4 g per 100 g of product; nominally approximately 9.6 mg·day^−1^ at the administered dose) was not independently verified, and the extraction ratio and standardized-extract status were not specified. The findings therefore apply specifically to the *Rhodiola rosea* product and dosing protocol used in this study. Seventh, all participants completed follow-up, but a group-specific numerical pill-adherence percentage was not retained; capsule distribution and completion of follow-up should not be interpreted as a quantified dose-adherence estimate. Eighth, blinding integrity was not formally assessed after the intervention. Ninth, the fixed 30 min CAF interval was shorter than the frequently used 45–60 min window, and the results should not be extrapolated to other administration intervals. Finally, registration was retrospective (30 June 2026) because the investigators initially misclassified the project as an exercise-performance and sports-nutrition study in healthy athletes rather than an interventional trial requiring prospective registration. The registry is therefore reported transparently as a retrospective record rather than evidence of prospective outcome specification. The non-significant CAF × RHO interaction indicates no evidence of interaction in this dataset, not proof that an interaction is impossible.

## 5. Conclusions

In male collegiate DanceSport athletes, baseline-adjusted ANCOVA identified group differences in all four registry-listed Jive-specific primary outcomes after Holm correction. Adjusted 30 s valid-kick success rates were 84.66% in CTR, 90.69% in CAF, 84.43% in RHO, and 92.31% in RHO + CAF; CAF and RHO + CAF were higher than CTR and RHO.

Most non-redundant secondary outcomes also met the false-discovery-rate criterion, whereas YBT did not. The factorial analysis showed no CAF × RHO interaction for the primary outcomes. The findings therefore support outcome-specific group differences without establishing synergy or a physiological mechanism.

## Figures and Tables

**Table 1:** Weekly Jive-specific training plan during the 4-week intervention.

Training Day	Session Duration (min)	Main Training Content	Main Focus
Monday	75	Basic Jive rhythm training, bouncing-rhythm drills, low-intensity kicking technique drills, and leg-retraction control	Maintenance of specific technique and movement standardization
Tuesday	75	Lower-limb explosive training, split-lunge squat and step-up training (4 sets × 12 repetitions each), repeated kicking practice, weight-transfer training, and core stability control training	Physical and neuromuscular demands related to rapid kicking and kick-retraction linkage
Thursday	75	30 s specific kicking training (8 eight-counts, 32 kicks), rhythm-maintenance training, trunk anteroposterior sway control, and specific endurance training	Continuous movement quality, body stability, and resistance to later-phase technical breakdown
Saturday	75	Jive routine-fragment practice, competition-scenario simulation, movement-quality maintenance, cool-down, and recovery activities	Integration of specific performance, competition rhythm adaptation, and fatigue management

Note: The standard unit of the specific kick task was 30 s, 8 eight-counts, and 32 total kicks. Split-lunge squat and step-up/box step were performed as 4 sets × 12 repetitions to strengthen rapid lower-limb extension, movement linkage, and later-phase rhythm maintenance.

**Table 2:** Participant baseline characteristics.

Variable	CTR (*n* = 12)	CAF (*n* = 12)	RHO (*n* = 12)	RHO + CAF (*n* = 12)
Age (years)	20.3 ± 1.7	20.8 ± 1.2	20.3 ± 1.7	20.3 ± 1.5
Height (cm)	177.4 ± 2.7	179.1 ± 2.2	179.1 ± 1.9	177.7 ± 2.8
Body mass (kg)	68.2 ± 5.8	66.3 ± 4.4	68.6 ± 4.6	67.1 ± 5.6
BMI (kg·m^−2^)	21.7 ± 2.0	20.7 ± 1.5	21.4 ± 1.4	21.3 ± 1.7
Training experience (years)	5.1 ± 1.7	6.1 ± 1.7	5.2 ± 1.1	5.1 ± 1.4

**Table 3:** Raw Pre/Post values and baseline-adjusted ANCOVA results for registry-listed outcomes.

Outcome	CTR Pre/Post	CAF Pre/Post	RHO Pre/Post	RHO + CAF Pre/Post	ANCOVA F (df)	Raw *p*	Adjusted *p*; Partial η^2^
Kick-extension angle [P]	176.73 ± 0.71/ 176.57 ± 0.82	176.79 ± 0.75/ 177.68 ± 1.20	176.72 ± 1.05/ 177.73 ± 0.79	176.75 ± 0.67/ 178.03 ± 1.15	4.722 (3,43)	0.006182	Holm 0.006182; 0.248
Post-kick leg-retraction angle [P]	59.93 ± 1.92/ 60.06 ± 1.66	60.08 ± 1.47/ 62.35 ± 1.80	59.86 ± 1.69/ 62.49 ± 2.18	59.92 ± 1.55/ 63.03 ± 2.00	5.515 (3,43)	0.002701	Holm 0.005401; 0.278
Trunk anteroposterior sway [P]	14.10 ± 1.28/ 14.02 ± 0.99	14.15 ± 0.96/ 12.83 ± 0.82	13.99 ± 0.87/ 12.83 ± 1.12	14.13 ± 0.86/ 12.54 ± 0.98	6.223 (3,40)	0.001432	Holm 0.004295; 0.318
30 s valid-kick success rate (%) [P]	83.33 ± 7.46/ 84.64 ± 6.17	83.59 ± 6.27/ 90.63 ± 4.62	83.07 ± 6.31/ 84.38 ± 5.96	83.07 ± 6.31/ 92.19 ± 4.52	8.382 (3,40)	0.000192	Holm 0.000768; 0.386
30 s valid-kick count [S, descriptive]	26.67 ± 2.39/ 27.08 ± 1.98	26.75 ± 2.01/ 29.00 ± 1.48	26.58 ± 2.02/ 27.00 ± 1.91	26.58 ± 2.02/ 29.50 ± 1.45	-	-	Exact numerator of success rate
YBT composite balance score (%) [S]	90.97 ± 2.26/ 91.00 ± 2.10	91.10 ± 2.62/ 91.48 ± 1.67	90.93 ± 2.40/ 90.65 ± 1.62	91.04 ± 2.42/ 91.18 ± 2.01	0.404 (3,43)	0.750784	BH 0.750784; 0.027
Bird-dog hold time (s) [S]	41.88 ± 2.38/ 42.07 ± 2.51	41.81 ± 2.60/ 44.97 ± 2.11	41.76 ± 1.91/ 42.10 ± 2.18	41.83 ± 2.06/ 45.26 ± 2.71	6.352 (3,43)	0.001161	BH 0.003734; 0.307
Split-lunge valid count (0–48) [S]	42.92 ± 1.78/ 43.08 ± 2.02	42.67 ± 2.53/ 45.25 ± 2.05	43.08 ± 2.64/ 45.67 ± 2.06	42.83 ± 2.52/ 46.08 ± 1.56	5.595 (3,43)	0.002490	BH 0.003734; 0.281
Step-up valid count (0–48) [S]	43.08 ± 1.88/ 43.00 ± 1.54	43.00 ± 2.13/ 45.33 ± 2.19	43.08 ± 2.02/ 45.50 ± 1.73	42.83 ± 2.66/ 45.67 ± 2.02	5.370 (3,43)	0.003138	BH 0.004035; 0.273
45 s high-knee compliance (%) [S]	86.43 ± 5.10/ 86.07 ± 3.86	85.93 ± 4.39/ 90.98 ± 4.45	85.99 ± 5.30/ 86.01 ± 4.92	86.34 ± 4.83/ 92.15 ± 3.83	6.644 (3,43)	0.000870	BH 0.003734; 0.317
505 change-of-direction time (s) [S]	4.68 ± 0.49/ 4.89 ± 0.47	4.70 ± 0.59/ 4.32 ± 0.51	4.59 ± 0.54/ 4.22 ± 0.44	4.69 ± 0.41/ 4.06 ± 0.57	6.058 (3,43)	0.001557	BH 0.003734; 0.297
Illinois agility time (s) [S]	20.37 ± 2.92/ 23.28 ± 2.79	20.67 ± 2.60/ 19.99 ± 2.57	21.11 ± 2.65/ 19.93 ± 2.20	21.22 ± 2.46/ 19.25 ± 2.74	5.683 (3,43)	0.002275	BH 0.003734; 0.284
Hexagon-jump time (s) [S]	16.63 ± 1.62/ 17.98 ± 2.06	16.85 ± 1.91/ 15.36 ± 2.47	16.43 ± 1.17/ 15.14 ± 2.01	16.18 ± 1.87/ 14.65 ± 2.05	5.604 (3,43)	0.002465	BH 0.003734; 0.281
Session-RPE [S]	7.66 ± 1.04/ 7.58 ± 0.99	7.53 ± 0.95/ 7.73 ± 0.84	8.01 ± 1.10/ 7.73 ± 1.16	7.33 ± 0.97/ 6.46 ± 0.91	4.563 (3,43)	0.007321	BH 0.008236; 0.241

Note: Values are mean ± SD. P, registry-listed primary outcome; S, registry-listed secondary outcome. Post values were analyzed by ANCOVA using the corresponding Pre value as covariate. Holm adjustment covered the four primary outcomes; Benjamini–Hochberg adjustment covered the nine non-redundant secondary outcomes. Valid-kick count is the exact numerator of success rate and was retained descriptively. Pairwise adjusted differences and 95% CIs are provided in Supplementary Data S1. Group × Pre interaction models were used for trunk anteroposterior sway and valid-kick success rate, with estimates evaluated at the overall Pre mean. Supplementary Data S1 provides all Bonferroni-adjusted pairwise contrasts, simultaneous 95% CIs, model diagnostics, HC3-robust sensitivity analyses, and primary-outcome factorial interaction estimates.

## Data Availability

De-identified participant data and supplementary statistical outputs are available from the corresponding author upon reasonable request, subject to ethics approval and data-management requirements. The participant-level allocation log and identifiable records are restricted to protect privacy.

## Supplementary Materials

The following supporting information can be downloaded at: https://www.mdpi.com/article/10.3390/nu18172823/s1, Figure S1. Representative field sequences for the registered secondary stability assessments. Figure S2. Representative field sequences for the registered secondary specific-strength and rhythm assessments. Figure S3. Representative field sequences for the registered secondary agility assessments. Data S1: Registry-aligned ANCOVA estimates, complete Bonferroni-adjusted pairwise contrasts with simultaneous 95% confidence intervals, model diagnostics, HC3-robust sensitivity analyses, and exploratory CAF × RHO interaction estimates for the primary outcomes. Product Documentation S1: Original product photographs and English-translated label information for the *Rhodiola rosea* product used in this study. Figure S4. (A) Original front-view photograph of the Tongrentang Brand Rhodiola Capsule; (B) Original side-view photograph showing ingredients, labeled use, specification, production date, batch number, and expiry date; (C) Original side/back-view photograph showing manufacturer, registration, production-license, product-standard, and barcode information.

## Abbreviations

The following abbreviations are used in this manuscript:

CAF	caffeine
RHO	tested Rhodiola capsule preparation
RHO + CAF	Rhodiola capsule preparation plus caffeine supplementation
CTR	placebo control group
Pre	baseline assessment
Post	post-intervention assessment
YBT	Y-Balance Test
RPE	rating of perceived exertion
session-RPE	session rating of perceived exertion
ANOVA	analysis of variance; ANCOVA: analysis of covariance
SD	standard deviation
CI	confidence interval
BH	Benjamini–Hochberg
CONSORT	Consolidated Standards of Reporting Trials

## References

[1] Koutedakis Y., Jamurtas A. (2004). The dancer as a performing athlete: Physiological considerations. Sports Med..

[2] Blanksby B.A., Reidy P.W. (1988). Heart rate and estimated energy expenditure during ballroom dancing. Br. J. Sports Med..

[3] Liiv H., Jurimae T., Maestu J., Purge P., Hannus A., Jurimae J. (2014). Physiological characteristics of elite dancers of different dance styles. Eur. J. Sport Sci..

[4] Wilson M., Kwon Y.H. (2008). The role of biomechanics in understanding dance movement: A review. J. Dance Med. Sci..

[5] Russell J.A. (2013). Preventing dance injuries: Current perspectives. Open Access J. Sports Med..

[6] Ngo J.K., Lu J., Cloak R., Wong D.P., Devonport T., Wyon M.A. (2024). Strength and conditioning in dance: A systematic review and meta-analysis. Eur. J. Sport Sci..

[7] Rodrigues-Krause J., Krause M., Reischak-Oliveira A. (2015). Cardiorespiratory considerations in dance: From classes to performances. J. Dance Med. Sci..

[8] Rodrigues-Krause J., Farinha J.B., Krause M., Reischak-Oliveira A. (2016). Effects of dance interventions on cardiovascular risk with ageing: Systematic review and meta-analysis. Complement. Ther. Med..

[9] Bria S., Bianco M., Galvani C., Palmieri V., Zeppilli P., Faina M. (2011). Physiological characteristics of elite sport-dancers. J. Sports Med. Phys. Fit..

[10] Liiv H., Wyon M., Jurimae T., Purge P., Saar M., Maestu J., Jurimae J. (2014). Anthropometry and somatotypes of competitive DanceSport participants: A comparison of three different styles. Homo.

[11] Guest N.S., VanDusseldorp T.A., Nelson M.T., Grgic J., Schoenfeld B.J., Jenkins N.D.M., Arent S.M., Antonio J., Stout J.R., Trexler E.T. (2021). International Society of Sports Nutrition position stand: Caffeine and exercise performance. J. Int. Soc. Sports Nutr..

[12] Grgic J., Grgic I., Pickering C., Schoenfeld B.J., Bishop D.J., Pedisic Z. (2020). Wake up and smell the coffee: Caffeine supplementation and exercise performance-an umbrella review of 21 published meta-analyses. Br. J. Sports Med..

[13] Southward K., Rutherfurd-Markwick K.J., Ali A. (2018). The effect of acute caffeine ingestion on endurance performance: A systematic review and meta-analysis. Sports Med..

[14] Warren G.L., Park N.D., Maresca R.D., McKibans K.I., Millard-Stafford M.L. (2010). Effect of caffeine ingestion on muscular strength and endurance: A meta-analysis. Med. Sci. Sports Exerc..

[15] Grgic J., Trexler E.T., Lazinica B., Pedisic Z. (2018). Effects of caffeine intake on muscle strength and power: A systematic review and meta-analysis. J. Int. Soc. Sports Nutr..

[16] Spriet L.L. (2014). Exercise and sport performance with low doses of caffeine. Sports Med..

[17] Pickering C., Kiely J. (2018). Are the current guidelines on caffeine use in sport optimal for everyone? Inter-individual variation in caffeine ergogenicity, and a move towards personalised sports nutrition. Sports Med..

[18] Mohr M., Nielsen J.J., Bangsbo J. (2011). Caffeine intake improves intense intermittent exercise performance and reduces muscle interstitial potassium accumulation. J. Appl. Physiol..

[19] Schneiker K.T., Bishop D., Dawson B., Hackett L.P. (2006). Effects of caffeine on prolonged intermittent-sprint ability in team-sport athletes. Med. Sci. Sports Exerc..

[20] Ferreira R.E.S., Pacheco R.L., de Oliveira Cruz Latorraca C., Riera R., Eid R.G., Martimbianco A.L.C. (2021). Effects of caffeine supplementation on physical performance of soccer players: Systematic review and meta-analysis. Sports Health.

[21] Gomez-Bruton A., Marin-Puyalto J., Muniz-Pardos B., Matute-Llorente A., Del Coso J., Gomez-Cabello A., Vicente-Rodriguez G., Casajus J.A., Lozano-Berges G. (2021). Does acute caffeine supplementation improve physical performance in female team-sport athletes? Evidence from a systematic review and meta-analysis. Nutrients.

[22] De Bock K., Eijnde B.O., Ramaekers M., Hespel P. (2004). Acute *Rhodiola rosea* intake can improve endurance exercise performance. Int. J. Sport Nutr. Exerc. Metab..

[23] Noreen E.E., Buckley J.G., Lewis S.L., Brandauer J., Stuempfle K.J. (2013). The effects of an acute dose of *Rhodiola rosea* on endurance exercise performance. J. Strength Cond. Res..

[24] Tinsley G.M., Jagim A.R., Potter G.D.M., Garner D., Galpin A.J. (2024). *Rhodiola rosea* as an adaptogen to enhance exercise performance: A review of the literature. Br. J. Nutr..

[25] Ishaque S., Shamseer L., Bukutu C., Vohra S. (2012). *Rhodiola rosea* for physical and mental fatigue: A systematic review. BMC Complement. Altern. Med..

[26] Lu Y., Deng B., Xu L., Liu H., Song Y., Lin F. (2022). Effects of *Rhodiola rosea* supplementation on exercise and sport: A systematic review. Front. Nutr..

[27] Schwarz N.A., Stratton M.T., Colquhoun R.J., Manganti A.M., Sherbourne M., Mourey F., White C.C., Day H., Dusseault M.C., Hudson G.M. (2024). Salidroside and exercise performance in healthy active young adults—An exploratory, randomized, double-blind, placebo-controlled study. J. Int. Soc. Sports Nutr..

[28] Dou Y., Wang Y., Zhang W., Jiang Y., Zhang J., Yang T., Han Z., Li Y., Liu C., Ren D. (2026). Effects of *Rhodiola rosea* on physical and decision-making performance in football players: A randomised controlled trial. Nutrients.

[29] Wang X., Yang X., Gao Z., Zeng J., Liu Y. (2025). The effect of *Rhodiola rosea* supplementation on endurance performance and related biomarkers: A systematic review and meta-analysis. Front. Nutr..

[30] Liu C., Zhao H., Yan Y., Yang W., Chen S., Song G., Li X., Gu Y., Yun H., Li Y. (2023). Synergistic effect of *Rhodiola rosea* and caffeine supplementation on the improvement of muscle strength and muscular endurance: A pilot study for rats, resistance exercise-untrained and -trained volunteers. Nutrients.

[31] Yun H., Lu B., Su W., Wang J., Zheng J., Wang J., Wang Z., Li Y., Sun Y., Liu C. (2024). Combined effects of *Rhodiola rosea* and caffeine supplementation on aerobic endurance and muscle explosiveness: A synergistic approach. Front. Nutr..

[32] Wang Z., Du H., Li H., Zhao K., Zhao B., Liu C. (2025). Effects of the Combined Supplementation of Caffeine and *Rhodiola rosea* with Resistance Training on Lower Limb Explosive Power in Male Volleyball Players. Nutrients.

[33] Dou Y., Feng Z., Xu H., Ma H., Jiang Y., Lyu X., Han B., Liu S., Liu C., Ren D. (2026). Effects of combined caffeine and *Rhodiola rosea* supplementation on repeated aerial duel performance and neck neuromuscular function in soccer players. Nutrients.

[34] De Pauw K., Roelands B., Cheung S.S., de Geus B., Rietjens G., Meeusen R. (2013). Guidelines to classify subject groups in sport-science research. Int. J. Sports Physiol. Perform..

[35] Harriss D.J., Jones C., MacSween A. (2022). Ethical standards in sport and exercise science research: 2022 update. Int. J. Sports Med..

[36] Hopewell S., Chan A.W., Collins G.S., Hrobjartsson A., Moher D., Schulz K.F., Tunn R., Aggarwal R., Berkwits M., Berlin J.A. (2025). CONSORT 2025 statement: Updated guideline for reporting randomised trials. BMJ.

[37] Plisky P.J., Rauh M.J., Kaminski T.W., Underwood F.B. (2006). Star Excursion Balance Test as a predictor of lower extremity injury in high school basketball players. J. Orthop. Sports Phys. Ther..

[38] Gribble P.A., Hertel J., Plisky P. (2012). Using the Star Excursion Balance Test to assess dynamic postural-control deficits and outcomes in lower extremity injury: A literature and systematic review. J. Athl. Train..

[39] Plisky P., Schwartkopf-Phifer K., Huebner B., Garner M.B., Bullock G. (2021). Systematic review and meta-analysis of the Y-Balance Test Lower Quarter: Reliability, discriminant validity, and predictive validity. Int. J. Sports Phys. Ther..

[40] Hachana Y., Chaabene H., Nabli M.A., Attia A., Moualhi J., Farhat N., Elloumi M. (2013). Test-retest reliability, criterion-related validity, and minimal detectable change of the Illinois Agility Test in male team sport athletes. J. Strength Cond. Res..

[41] Sheppard J.M., Young W.B. (2006). Agility literature review: Classifications, training and testing. J. Sports Sci..

[42] Barber O.R., Thomas C., Jones P.A., McMahon J.J., Comfort P. (2016). Reliability of the 505 Change-of-Direction Test in netball players. Int. J. Sports Physiol. Perform..

[43] Girard O., Mendez-Villanueva A., Bishop D. (2011). Repeated-sprint ability-Part I: Factors contributing to fatigue. Sports Med..

[44] Bishop D., Girard O., Mendez-Villanueva A. (2011). Repeated-sprint ability-Part II: Recommendations for training. Sports Med..

[45] Markovic G. (2007). Does plyometric training improve vertical jump height? A meta-analytical review. Br. J. Sports Med..

[46] Impellizzeri F.M., Rampinini E., Coutts A.J., Sassi A., Marcora S.M. (2004). Use of RPE-based training load in soccer. Med. Sci. Sports Exerc..

[47] Haddad M., Stylianides G., Djaoui L., Dellal A., Chamari K. (2017). Session-RPE method for training load monitoring: Validity, ecological usefulness, and influencing factors. Front. Neurosci..

[48] Niazi H., Benbaruj J., Sheel A.W., MacNutt M.J. (2026). Exercise physiology trails the field in sex and gender equity: A call for faster progress, higher standards, and stronger science. Appl. Physiol. Nutr. Metab..

