# Supplementary material for: Effects of Caffeine and Rhodiola rosea, Alone and Combined, on DanceSport-Specific Jive Performance: A Randomized, Blinded, Placebo-Controlled Trial
Source: Nutrients. 2026 Aug 28;18(17):2823. doi: 10.3390/nu18172823 (PMC13567381; doi:10.3390/nu18172823)
Supplement: Supplementary file 1 [file nutrients-18-02823-s001.zip › nutrients-4434605-supplementary.pdf]

Supplementary Materials

Effects of Caffeine and Rhodiola, Alone and Combined, on DanceSport-Specific Jive Performance

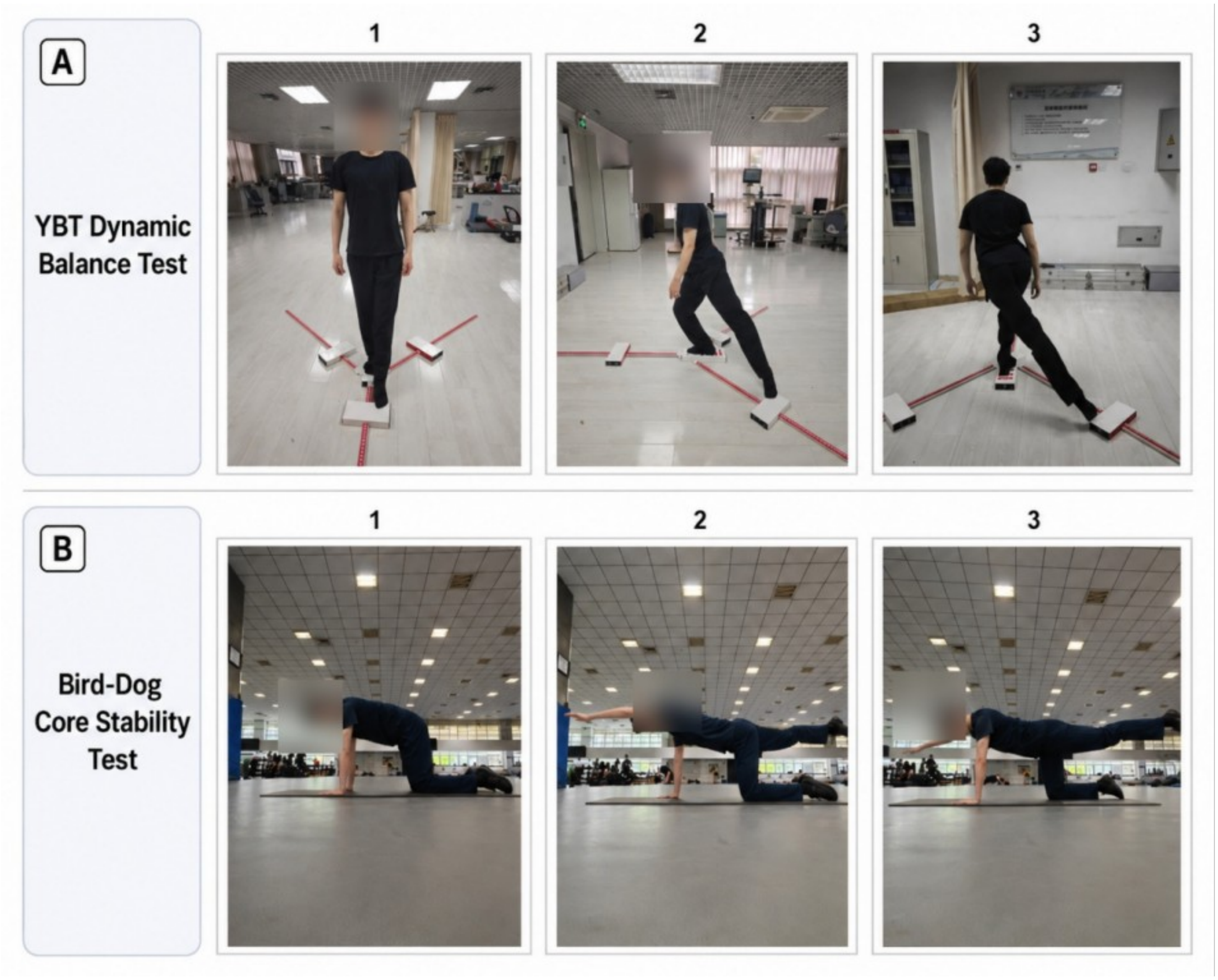

Supplementary Figure S1. Representative field sequences for the registered secondary stability assessments. (A1–A3) Y-Balance Test (YBT): starting position and multidirectional reach phases; (B1–B3) bird-dog core stability test: setup, contralateral arm–leg extension, and standardized hold. Images illustrate the standardized testing procedures only; quantitative outcomes were obtained using the scoring procedures described in the Methods. Faces were obscured to protect participant privacy.

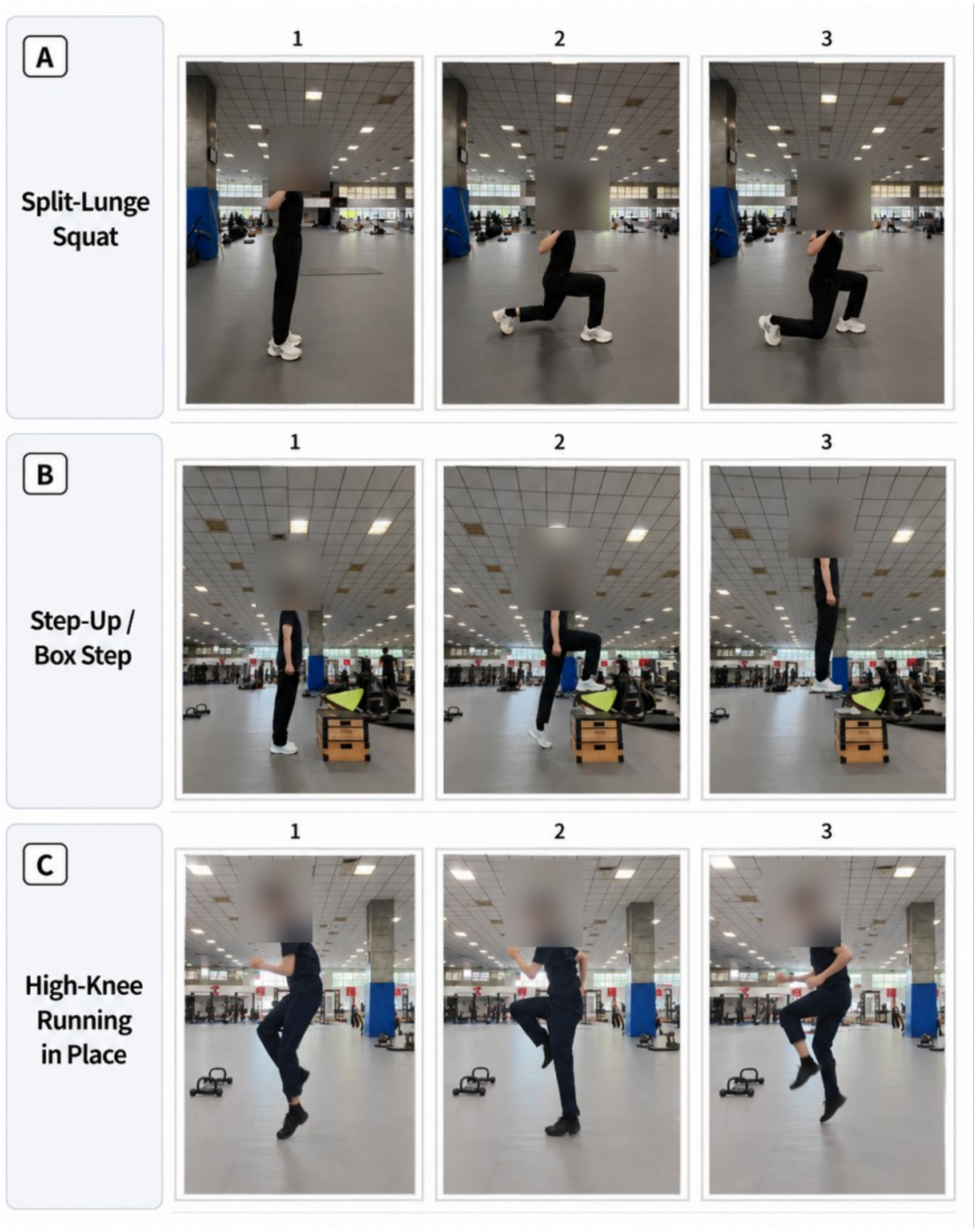

Supplementary Figure S2. Representative field sequences for the registered secondary specific-strength and rhythm assessments. (A1–A3) split-lunge squat; (B1–B3) step-up/box step; (C1–C3) 45-s high-knee running in place. The panels illustrate the setup, execution, and completion phases. Images are procedural illustrations and do not represent participant-level statistical outcomes. Faces were obscured to protect participant privacy.

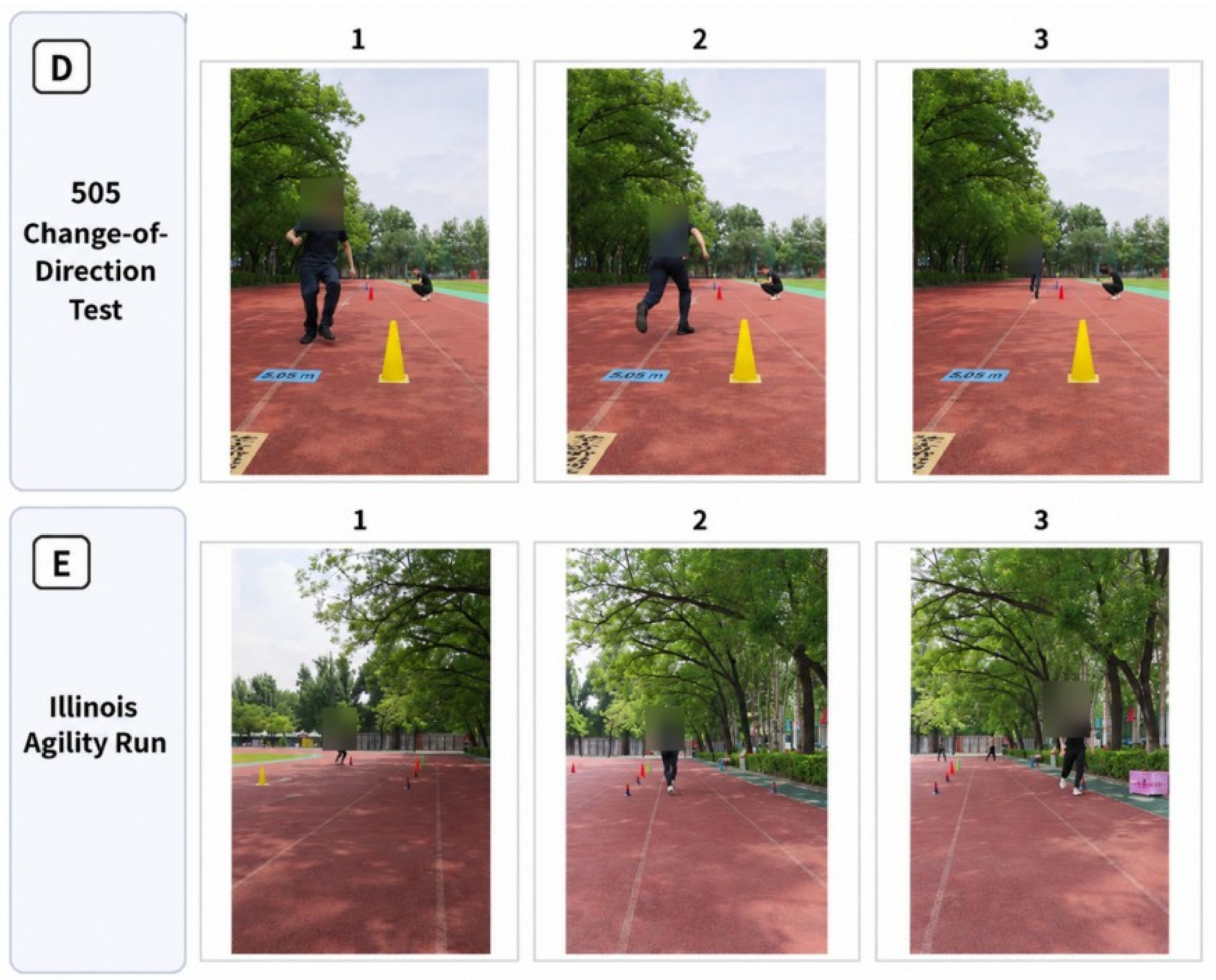

Supplementary Figure S3. Representative field sequences for the registered secondary agility assessments. (D1–D3) 505 change-of-direction test; (E1–E3) Illinois agility run. The panels illustrate the standardized test setup and movement sequence; timing outcomes were analyzed using the procedures described in the Methods. Faces were obscured to protect participant privacy.
