# Supplementary material for: Effects of Caffeine and Rhodiola rosea, Alone and Combined, on DanceSport-Specific Jive Performance: A Randomized, Blinded, Placebo-Controlled Trial
Source: Nutrients. 2026 Aug 28;18(17):2823. doi: 10.3390/nu18172823 (PMC13567381; doi:10.3390/nu18172823)
Supplement: Supplementary file 1 [file nutrients-18-02823-s001.zip › Supplementary_Product_Documentation_S1_EN_English_Only.pdf]

Supplementary Product Documentation S1

Original Product Photographs and English-Translated Label Information for the Rhodiola Product

S1. Product Identification

Product name: Tongrentang Brand Rhodiola Capsule

Manufacturer: Beijing Tongrentang Health Pharmaceutical Co., Ltd., Beijing, China

Chinese health-food registration no.: G20100243

Batch no.: 2025202010

Production date: 1 August 2025

Expiry date: 31 July 2027

S2. Product Label Information

Label item	**English translation**
Product name	Product name: Tongrentang Brand Rhodiola Capsule
Raw material	Raw material: Rhodiola (irradiated)
Excipients	Excipients: None
Characteristic constituent and content	Characteristic constituent: salidroside, 0.4 g per 100 g of product
Suitable population	Suitable population: individuals exposed to hypoxic environments
Not suitable for	Not suitable for: children, pregnant women, and lactating women
Health function	Health function: helps improve tolerance to hypoxia
Recommended intake and administration	Recommended intake: three capsules twice daily, orally
Specification	Specification: 0.4 g per capsule
Shelf life	Shelf life: 24 months
Storage	Storage: keep sealed in a cool, dry place
Production date	Production date: 1 August 2025
Batch number	Batch no.: 2025202010
Expiry date	Expiry date: 31 July 2027
Net content	Net content: 48 g (0.4 g × 120 capsules)
Product standard	Product standard: Q/DXTJP 0087J
Food production license number	Food production license no.: SC12711310100130
Precautions	Precautions: this product is not a substitute for medication; individuals outside the indicated population are not recommended to consume this product; this product does not provide supplemental oxygen.

S3. Study Administration

Participants assigned to RHO-containing conditions consumed three capsules twice daily, 30 min before breakfast and dinner, for four weeks. At 0.4 g per capsule, the administered product mass was 2.4 g/day.

Based on the label-declared salidroside content of 0.4 g per 100 g of product, the nominal label-derived daily salidroside intake was approximately 9.6 mg/day. The label-declared constituent content was not independently verified.

S4. Original Product Photographs

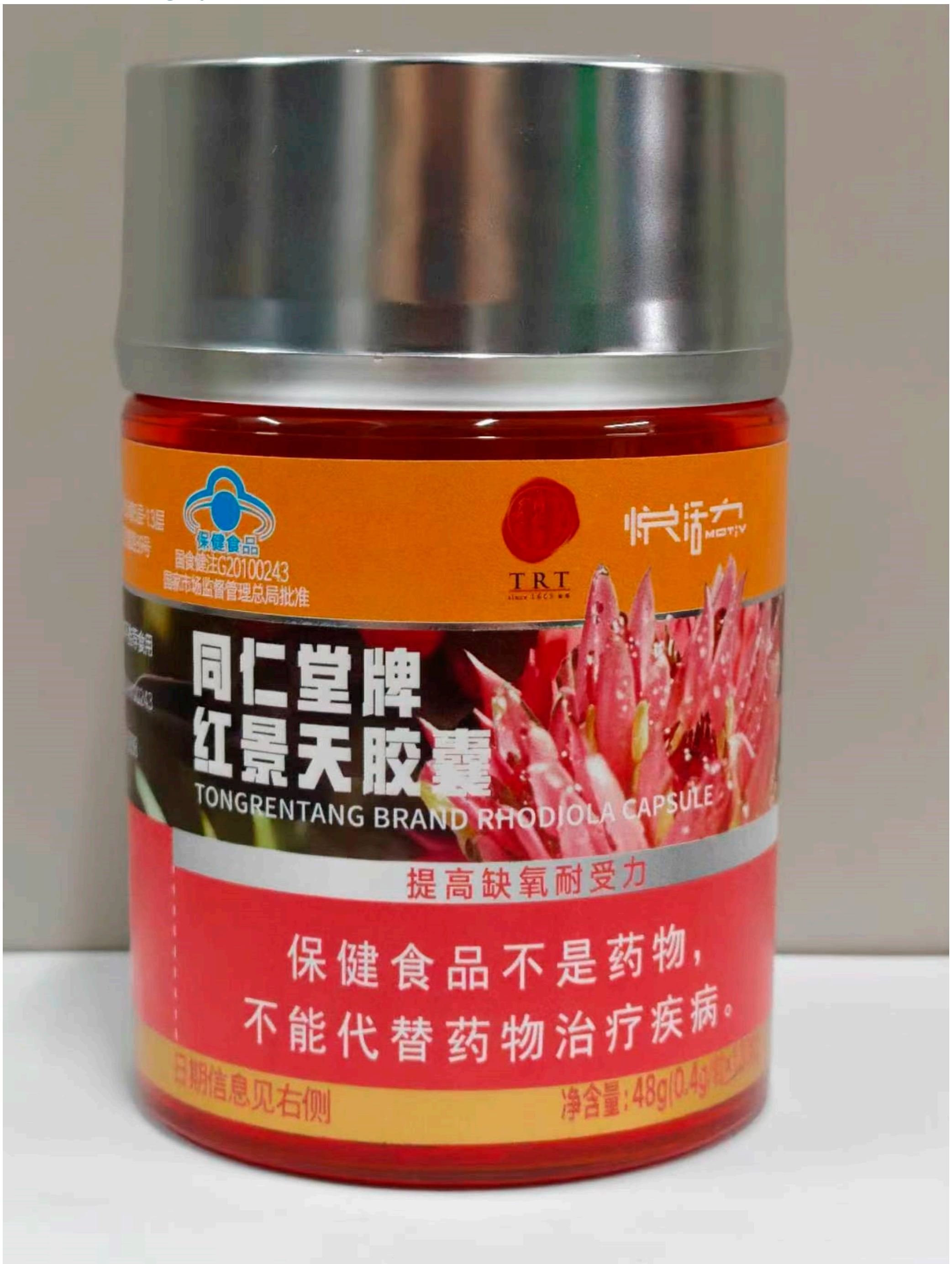

Figure S4A. Original front-view photograph of the Tongrentang Brand Rhodiola Capsule.

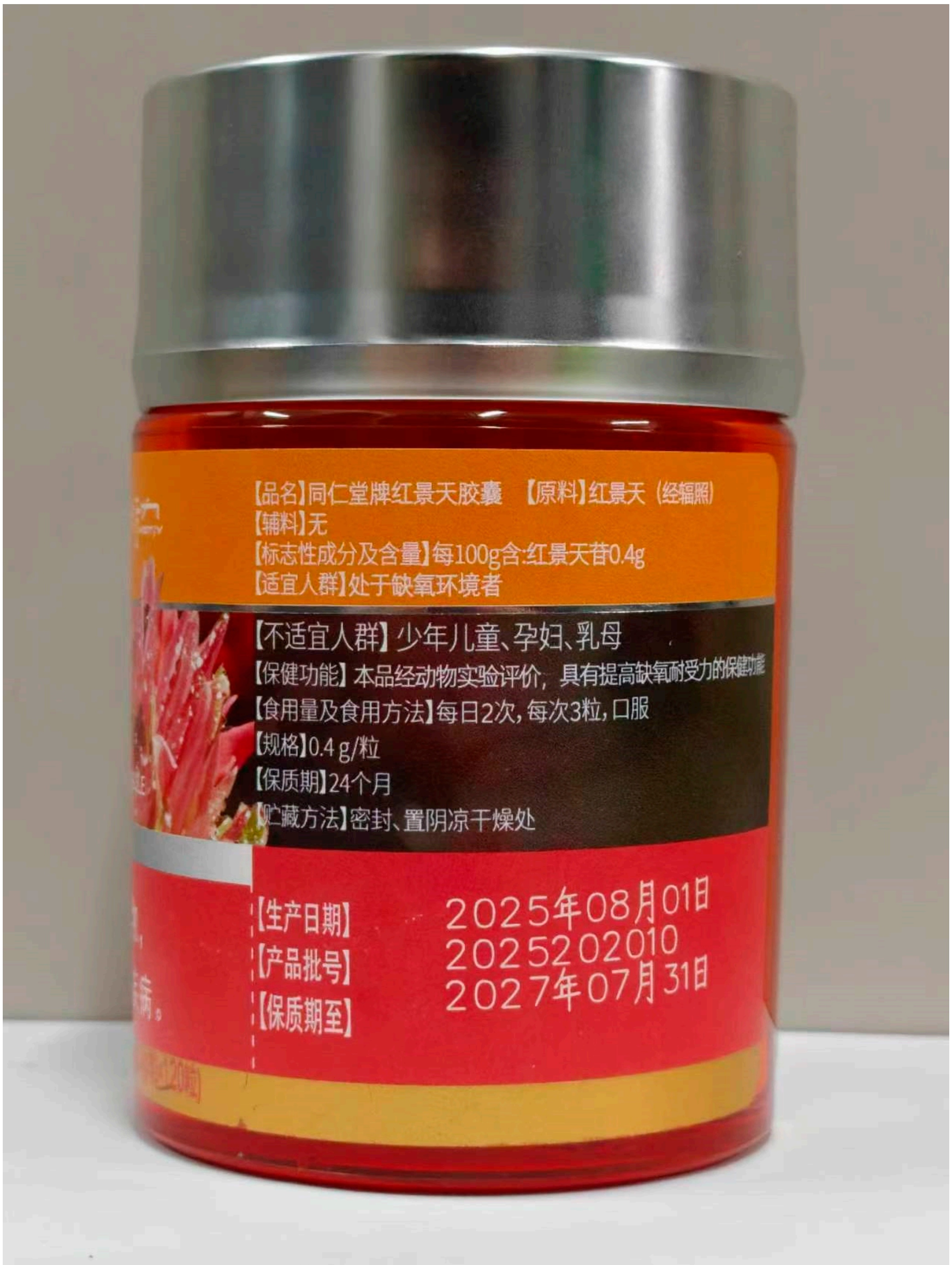

Figure S4B. Original side-view photograph showing ingredients, labeled use, specification, production date, batch number, and expiry date.

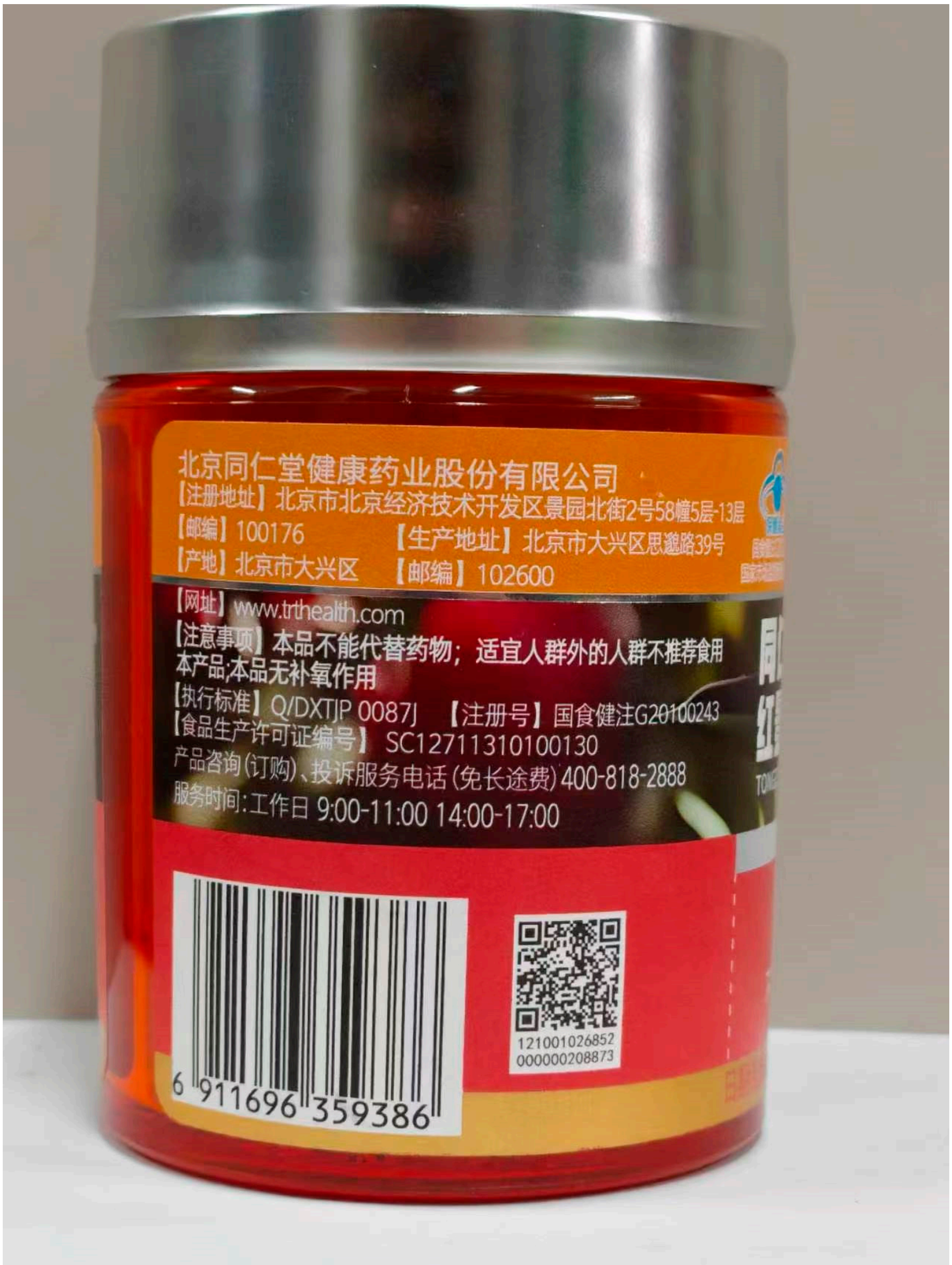

Figure S4C. Original side/back-view photograph showing manufacturer, registration, production-license, product-standard, and barcode information.

S5. Documentation Note

All information above was transcribed and translated from the original product packaging shown in Figures S1A–S1C. The label identifies the raw material as Rhodiola (irradiated). The label does not state a botanical species, extraction ratio, or standardized-extract status.
